# ApoA5 lowers triglyceride levels via suppression of ANGPTL3/8-mediated LPL inhibition

**DOI:** 10.1016/j.jlr.2021.100068

**Published:** 2021-03-21

**Authors:** Yan Q. Chen, Thomas G. Pottanat, Eugene Y. Zhen, Robert W. Siegel, Mariam Ehsani, Yue-Wei Qian, Robert J. Konrad

**Affiliations:** 1 Lilly Research Laboratories, Eli Lilly and Company, Indianapolis, IN, USA; 2 Department of Biology, Indiana University-Purdue University Indianapolis, Indianapolis, IN, USA

**Keywords:** LPL, triglycerides, angiopoietin-like protein, apolipoprotein, fatty acids, ANGPTL8, angiopoietin-like protein 8, HSA, human serum albumin, MSD, Meso Scale Discovery, SIL, stable isotope–labeled

## Abstract

Triglyceride (TG) molecules represent the major storage form of fatty acids, and TG metabolism is essential to human health. However, the mechanistic details surrounding TG metabolism are complex and incompletely elucidated. Although it is known that angiopoietin-like protein 8 (ANGPTL8) increases TGs through an ANGPTL3/8 complex that inhibits LPL, the mechanism governing ApoA5, which lowers TGs, has remained elusive. Current hypotheses for how ApoA5 acts include direct stimulation of LPL, facilitation of TG-containing particle uptake, and regulation of hepatic TG secretion. Using immunoprecipitation-MS and Western blotting, biolayer interferometry, functional LPL enzymatic assays, and kinetic analyses of LPL activity, we show that ApoA5 associates with ANGPTL3/8 in human serum and most likely decreases TG by suppressing ANGPTL3/8-mediated LPL inhibition. We also demonstrate that ApoA5 has no direct effect on LPL, nor does it suppress the LPL-inhibitory activities of ANGPTL3, ANGPTL4, or ANGPTL4/8. Importantly, ApoA5 suppression of ANGPTL3/8-mediated LPL inhibition occurred at a molar ratio consistent with the circulating concentrations of ApoA5 and ANGPTL3/8. Because liver X receptor (LXR) agonists decrease ApoA5 expression and cause hypertriglyceridemia, we investigated the effect of the prototypical LXR agonist T0901317 on human primary hepatocytes. We observed that T0901317 modestly stimulated hepatocyte ApoA5 release, but markedly stimulated ANGPTL3/8 secretion. Interestingly, the addition of insulin to T0901317 attenuated ApoA5 secretion, but further increased ANGPTL3/8 secretion. Together, these results reveal a novel intersection of ApoA5 and ANGPTL3/8 in the regulation of TG metabolism and provide a possible explanation for LXR agonist-induced hypertriglyceridemia.

Control of triglyceride (TG) metabolism to enable delivery of fatty acids (FAs) to target tissues such as muscle and fat involves a number of different proteins and is incompletely understood. Recently, the novel protein angiopoietin-like protein 8 (ANGPTL8) has garnered significant attention as a critical regulator of TG levels. ANGPTL8 was originally described several years ago as an atypical ANGPTL protein that increased TGs in an ANGPTL3-dependent manner, with knockout mice having decreased circulating TG and reduced fat mass (1, 2, 3, 4, 5, 6, 7). In 2016, Zhang described an elegant model for how ANGPTL8 worked with ANGPTL3 and ANGPTL4 to move FAs toward adipose tissue or skeletal muscle under feeding or fasting conditions, respectively (8). In 2017, Chi and colleagues demonstrated that ANGPTL8 enhanced the ability of ANGPTL3 to inhibit LPL by forming a complex that was required for its efficient secretion (9). In 2019, Kovrov *et al*. explored how ANGPTL8 could work with both ANGPTL3 and ANGPTL4 to partition FAs between adipose tissue and skeletal muscle (10).

We recently showed that the ANGPTL3/4/8 family of proteins is critical in regulating circulating TG levels through modulation of LPL activity in adipose tissue and skeletal muscle (11). In particular, we demonstrated that ANGPTL8 serves as the crucial insulin-responsive protein in this system by forming complexes with ANGPTL3 and ANGPTL4 to increase and decrease markedly their respective LPL-inhibitory activities. ANGPTL8 forms a circulating ANGPTL3/8 complex that dramatically increases ANGPTL3 inhibition of LPL in the skeletal muscle, resulting in increased circulating TGs that can be routed to the fat, where LPL inhibition is simultaneously decreased through formation of a localized ANGPTL4/8 complex. The localized ANGPTL4/8 complex also protects LPLs in the adipose tissue from circulating ANGPTL3/8 to ensure that the adipose tissue LPL is active after feeding. Together, these properties of ANGPTL8 result in increased LPL inhibition in the skeletal muscle and decreased LPL inhibition in the adipose tissue so that FAs are taken up into the fat postprandially and not deposited ectopically (11). Importantly, our work was subsequently corroborated with in vivo studies (12). As elegant as this remarkable system of proteins is, the above observations still cannot fully explain the control of TG metabolism, as several other proteins are known to influence TG concentrations. These include ApoC2 (which is thought to activate LPLs), ApoC3 (which is thought to inhibit LPL), and the atypical apolipoprotein, ApoA5 (13, 14, 15, 16, 17, 18, 19).

Interestingly, while there is clear agreement that ApoA5 potently decreases TG levels, its mechanism of action has remained obscure despite its discovery in 2001. The gene for ApoA5 was identified through experiments searching for open reading frames in the ApoA1-ApoC3-ApoA4 gene cluster located on human chromosome 11q23 (15, 16, 17, 18, 19). The new gene that emerged from this search coded for a novel apolipoprotein with greatest homology to ApoA4, and the corresponding apolipoprotein was appropriately named ApoA5. It soon became clear that ApoA5 was crucial in controlling circulating TG levels. When human ApoA5 was overexpressed in mice, it decreased TG concentrations by 50%–75%, and when the mouse ApoA5 gene was knocked out, TG concentrations increased approximately 4-fold (15, 16, 17, 18, 19). In addition, a number of ApoA5 mutations were reported in humans that correlated with circulating TGs (20, 21, 22, 23). It was also shown that ApoA5 mRNA expression was regulated by PPAR-α agonists and that administration of a PPAR-α agonist increased circulating ApoA5 levels, suggesting that this class of compounds may reduce serum TGs by increasing ApoA5 (24, 25, 26). Together, these initial observations clearly established ApoA5 as a critical player in TG metabolism.

Despite these compelling early data, however, no clear consensus has emerged for how ApoA5 actually acts at the molecular level to decrease TGs. Current hypotheses include suggestions that it may directly stimulate the LPL activity, facilitate TG-containing lipoprotein particle uptake by the liver, or intracellularly regulate the secretion of hepatic TG (27, 28, 29). One potential clue that the mechanism of action might be unusual came when we measured human serum levels of ApoA5 and found that it circulated as a 39-kD monomer at levels of 24–406 ng/ml, which are much lower than those of other apolipoproteins (30, 31). To put this in perspective, the molar concentration of ApoA5 is approximately 6 nM, compared with approximately 40 μM for ApoA1 and 15 μM for ApoC3. Yet, when transgenic mice had both ApoA5 and ApoC3 either knocked out or overexpressed, the result was essentially normal TG concentrations, although ApoC3 levels during overexpression were approximately 500-fold higher than those of overexpressed ApoA5, thus confirming the ability of ApoA5 to reduce serum TGs (19).

In light of these observations regarding ApoA5 as well as our own and other groups' recent studies on the ANGPTL3/4/8 system of proteins (8, 9, 10, 11), we sought to understand better the possible connections between these two important regulators of TG metabolism. In our present study, we demonstrate that ApoA5 associates with ANGPTL3/8 in human serum and present evidence that ApoA5 acts selectively to suppress the LPL-inhibitory activity of the ANGPTL3/8 complex. Using functional LPL assays, we show that ApoA5 has no direct effect on LPL activity and that it does not affect the LPL-inhibitory activities of ANGPTL3, ANGPTL4, or the ANGPTL4/8 complex. Importantly, we also demonstrate that the suppression of ANGPTL3/8-mediated LPL inhibition occurs at a molar ratio consistent with ApoA5 and ANGPTL3/8 concentrations in human serum. Upon obtaining these data, we also considered reports of liver X receptor (LXR) agonists decreasing ApoA5 expression and causing increases in TGs (32, 33). We observed that the prototypical LXR agonist T0901317 actually caused modest increases in hepatocyte ApoA5 secretion but markedly stimulated ANGPTL3/8 secretion. We also observed that the addition of insulin to T0901317 attenuated ApoA5 secretion while further increasing T0901317-stimulated ANGPTL3/8 secretion. Taken together, our results shed light on a novel intersection of ApoA5 and the ANGPTL3/4/8 family of proteins in the regulation of TG metabolism and also provide a possible explanation for LXR agonist–induced hypertriglyceridemia.

## Materials and methods

### Generation of recombinant ANGPTL proteins and complexes and ApoA5 protein

Human ANGPTL3, ANGPTL4, ANGPTL3/8, and ANGPTL4/8 were expressed as described previously (11). Mouse ANGPTL3/8 (mouse ANGPTL3: NP_038941.1 and mouse ANGPTL8: NP_001074409.1) was expressed utilizing the same techniques used for human ANGPTL3/8 (11). For ApoA5, the nucleotide sequences encoding *1*) human ApoA5 (NP_443200.2)-PreScission cleavage site-mature human serum albumin (HSA)-HIS tag, *2*) mouse IgG kappa signal peptide-HIS tag-mature HSA-PreScission cleavage site-mature human ApoA5 (residues 24–366), and *3*) mouse IgG kappa signal peptide-mature HSA-HIS tag were each inserted into a mammalian expression vector containing a cytomegalovirus promoter. Protein expression was performed through transient transfection in HEK293 cells cultured in the serum-free media. The culture media were harvested 5 days after transfection and stored at 4 °C for subsequent protein purification at 4 °C. Two liters of culture media were supplemented with 1 M Tris HCl (pH 8.0) and 5 M NaCl to final concentrations of 25 mM and 150 mM, respectively. The media were then incubated with 20 ml of HisPur Ni-NTA resin (Thermo) overnight. The resin was packed into a column and washed with buffer A (50 mM Tris HCl, 0.3 M NaCl, pH 8.0), and elution was performed with a 0–300 mM imidazole gradient in buffer A. Fractions containing the protein of interest were pooled, concentrated, and loaded onto a HiLoad Superdex 200 column (GE Healthcare) and eluted with PBS. Fractions of interest were pooled, protein concentrations were determined using a bicinchoninic acid protein assay, and the purified proteins were stored at -80 °C in PBS. In subsequent experiments, ApoA5 was either used as an HSA-tagged protein (in which case ApoA5 is stabilized with albumin), or in cases where the HSA tag was PreScission-cleaved, the nontagged ApoA5 protein was used immediately after cleavage. By performing experiments with ApoA5 in this manner, we avoided the use of stabilizers that could potentially have confounding effects on subsequent experiments performed with the protein. All proteins were maintained at a <0.01 EU/ug of endotoxin. Each protein (0.5 μg) was characterized with or without PreScission cleavage using gradient gel electrophoresis with Bio-Rad 4%–20% Mini-Protean Tris-glycine gels, followed by Coomassie Blue staining.

### Anti-ApoA5 and anti-ANGPTL protein/complex antibodies

ApoA5 antibodies were generated as described previously (30). Briefly, peptides corresponding to N-terminal and the C-terminal regions of human ApoA5 were synthesized. Terminal cysteine residues were added for conjugation to carrier proteins and chromatography beads, and peptides were conjugated to activated carriers. Rabbits were immunized every 21 days with 100 μg of peptide carrier conjugates in Freund's adjuvant and bled 10 days after booster injections beginning after the third injection. Polyclonal anti-C-terminal antisera were pooled, and polyclonal anti-N-terminal antisera were pooled. Afterward, the antibodies were affinity-purified against their respective peptides, concentrated with an Amicon stirred cell concentrator. Anti-human ANGPTL8 (residues 22–198) and anti-human ANGPTL3 (residues 17–220) antibodies were generated as described previously (11). Antibodies were biotinylated using a Pierce kit and ruthenium-labeled using a Meso Scale Discovery (MSD) kit, with MALDI-TOF performed to verify appropriate labeling. Anti-human ANGPTL3/8 complex–specific antibodies were generated via phage display or by immunizing mice with a recombinant ANGPTL3/8 complex using hybridoma techniques. Clones of interest were screened for nonoverlapping epitopes, and antigen-specific variable heavy (VH) and light (VL) gene sequences were determined from extracted RNA. Variable domains were transferred into separate constant region expression vectors for antibody production, transfected into CHO cells, and purified using protein A chromatography. Antibodies were tested for specificity against ANGPTL3/8 complex versus ANGPTL3 or ANGPTL8 using biolayer interferometry to select an anti-ANGPTL3/8 complex-specific antibody. All antibodies were diluted in 50% glycerol and stored at −20 °C.

### Identification of ANGPTL3/8-associated proteins by MS

Biotinylated anti-FLAG antibody in PBS was first captured on streptavidin-coated 96-well plates (Thermo). After washing away unbound antibody with PBS, ANGPTL3/8 complex containing a FLAG-tag sequence at the C terminus of ANGPTL3 was added to selected wells for 2 h at room temperature with gentle shaking. For control samples, no ANGPTL3/8 was added. Afterward, wells were washed with PBS, and 200 μl of pooled human serum (diluted 1:2 in PBS) were added to each well, with each sample analyzed in triplicate. The plate was then incubated at 4 °C overnight with gentle shaking to allow serum proteins to bind to the immobilized ANGPTL3/8 complex. The next day, the plate was washed 10 times using ice-cold PBS, and bound proteins were eluted using 100 μl of 1% acetic acid. The eluate was dried under nitrogen to remove acetic acid, and proteins were digested overnight at 37 °C using trypsin, after reduction and alkylation using triethylphosphine and iodoethanol, respectively (34). Digested peptides were desalted using micro Ziptips.

Samples were analyzed with a Thermo Q Exactive HF-X mass spectrometer using a Thermo Easy 1200 nLC-HPLC system. Peptide separation was carried out with a 75-μm × 15-cm Easy-Spray PepMap C18 column (Thermo) coupled to a Thermo Easy-Spray source. Solvent A was 0.1% formic acid in water (Thermo Fisher Scientific, Optima™ LC/MS grade), and solvent B was 80% acetonitrile with 0.1% formic acid (Thermo, Optima™ LC/MS grade). The gradient was 35 min using a flow rate of 250 nl/min, starting with a 32-min 5%–45% B ramp, followed by a 1-min 45%–95% B ramp, and a 2-min hold at 100% B. The HF-X was run with the following settings: spray voltage: 1.9 kV; capillary temperature: 275 °C; full scan at 120,000 resolution with AGC target at 3e6, max IT of 50 ms; dd-MS2 at 30,000 resolution with AGC target of 1e5, max IT of 100 ms, loop count of 20, CE of 25, isolation window of 2.0 m/z. Peptide sequences were identified by searching MS/MS spectra against the NCBI human database (35).

### MS assessment of ApoA5/ANGPTL3/8 complexes in human serum

The immunoprecipitation and MS techniques used for our analyses are similar to those described previously (11). Briefly, proteins were immunoprecipitated from 1 ml of pooled normal human serum using an anti-ANGPTL3/8 specific antibody and an irrelevant IgG as a negative control. Proteins were digested directly on-beads using Trypsin/Lys-C (Promega) after reduction and alkylation. Stable isotope–labeled (SIL) peptides (0.25 pmole) were spiked into each sample before analysis with a TSQ Quantiva (Thermo) using LC-multiple reaction monitoring (LC–MRM). For protein quantification, two SIL peptides for ANGPTL3, ANGPTL8, and ApoA5 were synthesized with selected lysine or arginine residues labeled with ^13^C and ^15^N, and the peptide content of SIL peptides was determined through amino acid analysis. Peak area ratios between the endogenous and corresponding SIL peptides were used to estimate protein concentrations after averaging results from two peptides for each protein analyzed. The specific SIL peptides used for quantitation are listed in Supplemental Tables.

### MS experimental design and rationale

Peptides with robust ionization after tryptic digest analyses of ANGPTL3/8 and ApoA5 were selected for MRM experiments. Protein BLAST (https://blast.ncbi.nlm.nih.gov/Blast.cgi) searches against the NCBI human database (the program's default) confirmed that these peptides were unique to the corresponding protein, with no known posttranslational modifications. At least two transitions were selected for each peptide for MRM. The amino acid sequences of these peptides and specific transitions are listed in Supplemental Tables. The transitions selected, the collision energy, and RF lens values were optimized by infusing the synthesized SIL peptides. No significant interference was detected at the corresponding retention time for each peptide in the negative control samples.

### Binding assessments

The interactions of the anti-ANGPTL3/8-specific antibody with ANGPTL3, ANGPTL8, and ANGPTL3/8 and the interaction of ANGPTL3/8 with ApoA5 were assessed with biolayer interferometry using Octet RED96e® (Molecular Devices). To evaluate the specificity of the anti-ANGPTL3/8 antibody for the ANGPTL3/8 complex versus free ANGPTL3 or ANGPTL8, the antibody was immobilized on streptavidin biosensors, incubated with ANGPTL3, ANGPTL8, or ANGPTL3/8 (100 nM each) and transferred into buffer-only wells to monitor dissociation. To study the interaction of ANGPTL3/8 with ApoA5, ANGPTL3/8 complex was first immobilized on biosensors with an anti-FLAG antibody. ANGPTL3/8 was then incubated with ApoA5 (6.25–100 nM HSA-ApoA5) to monitor association of ApoA5 with ANGPTL3/8 and then transferred into buffer-only wells to monitor its dissociation, with background reference subtraction. Curve fitting was performed using a 1:1 binding model to estimate k_on_, k_off_, and K_D_. To evaluate the effect of ApoA5 preincubation with ANGPTL3/8 on the interaction of ANGPTL3/8 with different antibodies, ANGPTL3/8 (24 nM) was preincubated with ApoA5 (0 or 2000 nM) at 37 °C for 1 h before binding analyses using biotinylated anti-ANGPTL3/8, anti-ANGPTL3, and anti-ANGPTL8 antibodies that had each been previously immobilized to streptavidin biosensors.

### Immunoprecipitation and Western blotting

The anti-ANGPTL3/8 antibody, an anti-ANGPTL3 antibody, an anti-ANGPT8 antibody, an anti-ApoA5 C-terminal antibody, and an irrelevant, negative control antibody were each covalently coupled to Tosyl-activated M-280 Dynabeads (Thermo), with heavy and light chains further cross-linked using dimethyl pimelimidate. For each immunoprecipitation, 50 μl of beads containing 20 μg of antibody were added to 2 ml of pooled donor serum (diluted 1:2 in PBS) and incubated at 4°C overnight. The following day, beads were washed with PBS and boiled in the sample buffer. Proteins were separated on a Novex 12% Bis-Tris gels and transferred to PVDF blots using an iBlot system (Thermo). Blots were probed with the selected biotinylated antibody of interest, and bands were visualized with Alexa Fluro 680–conjugated streptavidin using an Odyssey CLx image system (LI-COR Biosciences).

To verify that the anti-ANGPTL3/8 antibody pulled down ANGPTL3 and ANGPTL8, the anti-ANGPTL3/8 antibody was used to immunoprecipitate human serum, and bound proteins were analyzed by Western blotting with anti-ANGPTL3 and anti-ANGPTL8 antibodies. To show that the anti-ANGPTL3 and anti-ANGPTL8 antibodies pulled down ApoA5, the antibodies were used to immunoprecipitate human serum, and bound proteins were analyzed by Western blotting with an anti-ApoA5 antibody (as well as with anti-ANGPTL3 and anti-ANGPTL8 antibodies). In these experiments, immunoprecipitation of human serum with anti-ApoA5 antibody served as the positive control for pull down of ApoA5. To confirm that the anti-ANGPTL3/8 antibody pulled down ApoA5, the antibody was used to immunoprecipitate human serum, and bound proteins were analyzed by Western blotting with the anti-ApoA5 antibody. In these experiments, immunoprecipitation of human serum with an anti-ApoA5 antibody again served as the positive control for ApoA5 pull down. In all of the above experiments, the irrelevant antibody served as the negative control for immunoprecipitation, and recombinant ANGPTL3, ANGPTL8, or PreScission-cleaved HSA-ApoA5 loaded directly onto the gels served as the positive controls for Western blotting.

### ANGPTL3/8 and ApoA5 immunoassays

ANGPTL3/8 was measured as described previously (11). ApoA5 was also measured as previously described with minor modifications, including the use of HSA-ApoA5 for the calibration curve (30). For performing the ApoA5 assay, MSD streptavidin plates were washed with Tris-buffered saline containing 10 mmol/L Tris, pH 7.40, 150 mmol/L NaCl, and 1 ml/L Tween 20 and blocked with TBS plus 1% BSA for 1-h at room temperature. After aspiration and washing, wells were incubated with biotinylated anti–N-terminal ApoA5 capture antibody for 1-h. After washing, 50 μl of recombinant HSA-ApoA5 (serially diluted as a standard curve) was added to the wells in the assay buffer (50 mmol/L Hepes, pH 7.40, 150 mmol/L NaCl, 10 ml/L Triton X-100, 5 mmol/L EDTA, and 5 mmol/L EGTA). Samples were diluted in the assay buffer and added to their respective wells for a 2-h room temperature incubation. Wells were washed, and 50 μl of ruthenium-labeled anti-C-terminal ApoA5 detection antibody was added for a 1-h room temperature incubation. After washing of the wells, 150 μl of MSD read buffer was added. Electrochemiluminescence of ruthenium was detected using an MSD plate reader.

### Human LPL-stable expression cell line and activity assays

The nucleotide sequence for human LPL (NP_000228.1) was inserted into pLenti6.3 vector (Invitrogen) to generate lentivirus, which was used to create a stable expression cell line confirmed by qPCR and enzymatic activities. The cell line was maintained in DMEM/F12 (3:1) (Invitrogen), 10% FBS (Hyclone), and 5 μg/ml blasticidin (Invitrogen). The wild-type human LPL-stable expression cells were seeded at a density of 50,000 cells/well in tissue culture–treated 96-well plates (Costar) in the growth medium (3:1 DMEM/F12, 10% FBS, and 5 μg/ml blasticidin). After overnight incubation, the medium was replaced with 80 μl of the medium containing serially diluted ANGPTL proteins or complexes (that were previously preincubated for 1 h at 37°C in the absence or presence of ApoA5). Cells were incubated for 1 h before 20 μl of 5X working solution, freshly prepared with 0.05% Zwittergent detergent 3-(N,N-dimethyloctadecylammonio)-propanesulfonate (Sigma) and containing EnzChek lipase substrate BODIPY-dabcyl–labeled TG analog (Invitrogen), was added to achieve a final concentration of 1 μM. Fluorescence was monitored at 1 and 30 min with a Synergy Neo2 plate reader with an excitation wavelength of 485 nm and emission wavelength of 516 nm to correct for background. Kinetic analyses of the LPL activity were performed with modifications to allow for continuous characterization of LPL-inhibitory activities of ANGPTL proteins and complexes in the absence or presence of ApoA5. After overnight incubation of LPL-stable expression cells in growth medium, the medium was replaced with 80 μl of the medium containing ANGPTL proteins or complexes (preincubated in the absence or presence of ApoA5). Cells were incubated for 1 h at 37 °C before 20 μl of 5X working solution was added to achieve a final concentration of 1 μM substrate. The incubation was continued at 37 °C for 1 h, and fluorescence was monitored every minute.

### Mouse LPL-stable expression cell line and activity assays with mouse ANGPTL3/8 complex

The nucleotide sequence for mouse LPL (NP_032535.2) was used to create a stable expression cell line in a similar manner to the human LPL-stable cell line described above. After overnight incubation of the cells, the medium was replaced with 80 μl of the medium containing mouse ANGPTL3/8 complex (preincubated for 1 h at 37 °C in the absence or presence of human ApoA5). Cells were incubated for 1 h at 37 °C before 20 μl of 5X working solution was added to achieve a final concentration of 1 μM substrate. The incubation was continued at 37 °C for 1 h, and fluorescence was monitored every minute.

### LPL activity assay with VLDL as a substrate

The assay was similar to that described above, except that EnzChek lipase substrate was replaced with 20 μl/well of (Lee Solutions) VLDL (2 mM TG, final 0.4 mM TG in the well), and NEFA released by LPL were measured using an NEFA-HR kit (Wako). Human LPL-stable expression cells were seeded at 50,000 cells/well in a poly-D-lysine–coated 96-well plate. Cells were grown overnight, and the medium was replaced with 80 μl of the medium containing serially diluted ANGPTL proteins or complexes in the absence or presence of ApoA5 with 1-h preincubation at 37 °C. Cells were incubated for 1 h with gentle shaking. Twenty microliters of VLDL (2 mM TG) was added to each well, and the plate was incubated for 30 min, after which 20 μl of the medium was used for NEFA measurement.

### Secretion of ANGPTL3/8 and ApoA5 from hepatocytes

Human primary hepatocytes were obtained from BioIVT in the HepatoPac platform and were incubated for two days in the BioIVT maintenance media (11). After aspiration, cells were washed in serum-free BioIVT application media. Afterward, cells were preincubated for one day in the application media in the absence of insulin and then incubated overnight with the application media in the absence or presence of 0–1 nM insulin and/or 0–10,000 nM of the LXR agonist T0901317. Media were collected and stored at −80 °C before subsequent analyses of secreted ApoA5 and ANGPTL3/8 via their respective immunoassays.

### Statistics

A four-parameter logistic nonlinear regression model was used to fit LPL activity assay curves. For kinetic analyses of LPL assays, fluorescence was recorded every minute, and counts were plotted versus time to show the degree of LPL inhibition. For ANGPTL3/8 and ApoA5 immunoassays, MSD software was used for fitting of the immunoassay calibration curves using a 5-parameter fit with 1/y^2^ weighting. For MS data, a Student's *t* test was used to compare the log_2_ mean area under the curve (AUC) of the three replicates from control and ANGPTL3/8 samples. For ions with an extract AUC below 1024, the log_2_ AUC was adjusted to the log_2_ of 10 before statistical analysis. Peptide ions in ANGPTL3/8 samples differing from the control samples with a *P* < 0.05 and a fold-change > 5 were considered to be significant. Significance for the effect of insulin and T0901307 on hepatocyte ANGPTL3/8 and ApoA5 secretion was calculated using a one-way ANOVA. Significance for the combined effect was calculated using a two-way ANOVA.

## Results

### Association of ApoA5 with ANGPTL3/8 complex in human serum

**Fig. 1:**
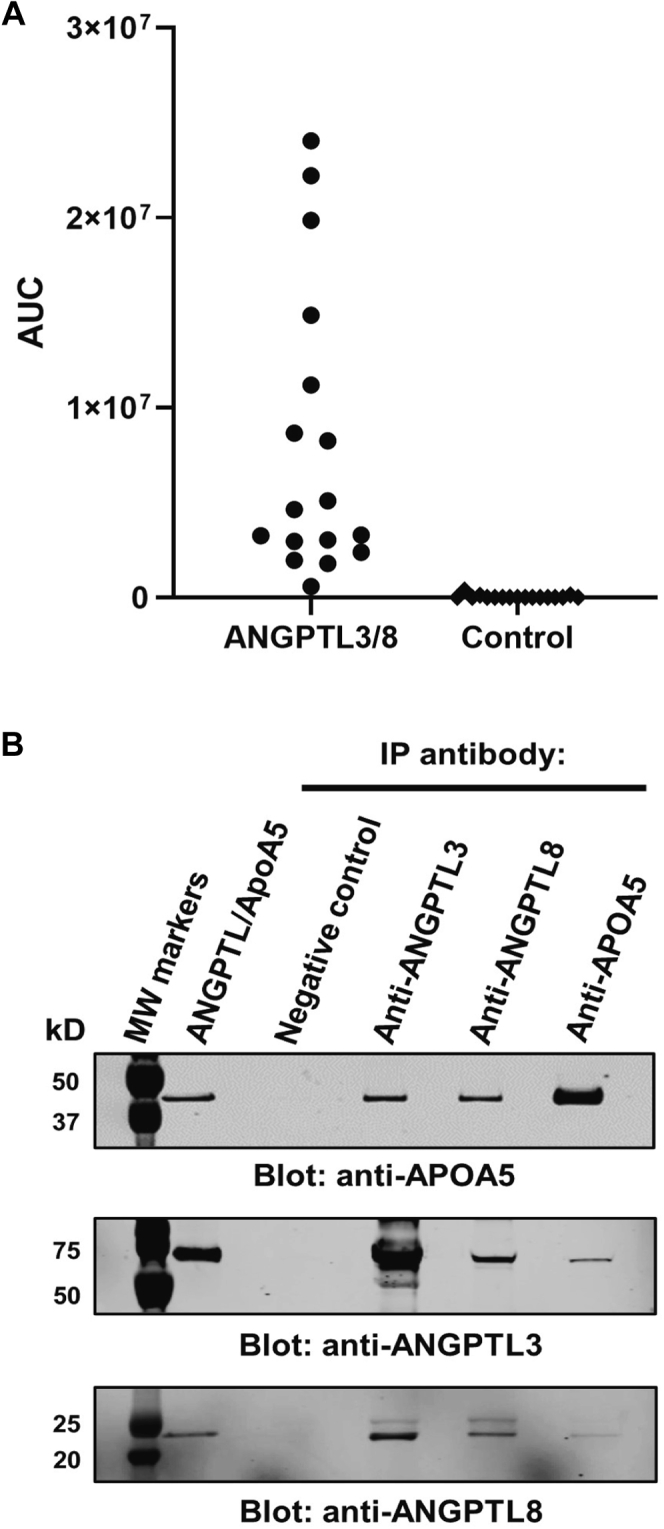
ApoA5 associates with ANGPTL3/8 in human serum. A: The ANGPTL3/8 complex was coupled to beads that were incubated with human serum overnight at 4°C. Bound proteins were reduced, alkylated, and digested with trypsin. The means (from triplicate samples) of the areas under the curve (AUC) for peptide ions significantly different from control (*P* < 0.05 and fold-change > 5) were plotted in a scatter dot plot. Only ApoA5 peptide ions met these criteria. The AUC was extracted for each ion with a mass tolerance of 2 ppm. Results shown are representative of 3 independent experiments. B: To confirm the association of ApoA5 with ANGPTL3/8 in human serum, an irrelevant, negative control antibody, an anti-ANGPTL3 antibody, an anti-ANGPTL8 antibody, and an anti-ApoA5 antibody were covalently coupled to beads and used to immunoprecipitate human serum overnight at 4°C. Immunoprecipitated proteins were separated on gels and transferred to PVDF membranes. The blots were stained with an anti-ApoA5 antibody, an anti-ANGPTL3 antibody, or an anti-ANGPTL8 antibody. ANGPTL8, angiopoietin-like protein 8.

To determine if circulating proteins associate with ANGPTL3/8, we incubated immobilized ANGPTL3/8 complex with human serum. After washing, bound proteins were eluted, reduced, and alkylated before trypsin digestion. Tryptic peptides were separated chromatographically, and the MS/MS spectra of each peptide were searched against a human database. Seventeen peptide ions were positively identified from ApoA5, and these 17 ions contained 12 unique peptide sequences covering 52% of the protein sequence. The mean AUCs for these 17 peptide ions from ANGPTL3/8-coated wells and control wells are shown in Fig. 1A. Database search results, database search settings, and the mean AUC for the ApoA5 peptide ions are listed in Supplemental Table S1. Extracted ion chromatograms are shown in Supplemental Figs. S1–S17. Of the 17 ApoA5 ions, 3 ions present at a very low level also registered positive AUC values in the control samples when extracting with a 2-ppm mass tolerance window. Detailed analyses, however, revealed that these were interference ions with a different isotope pattern compared with the ApoA5 ions.

Statistical analysis revealed that 9 of the 17 ions of ApoA5 were enriched greater than 5-fold (*P* < 0.001) than the control. Because ApoA5 was the only protein enriched greater than 5-fold than the control, these results indicated a prominent association of ApoA5 with ANGPTL3/8 in human serum, although we could not rule out the possibility of additional proteins being associated with ANGPTL3/8 in human serum. To verify the association of ApoA5 with ANGPTL3/8, we coupled anti-ANGPTL3, anti-ANGPTL8, and anti-ApoA5 antibodies to beads, incubated the beads with human serum, separated bound proteins via electrophoresis, transferred the proteins to PVDF, and performed Western blotting with anti-ANGPTL3, anti-ANGPTL8, and anti-ApoA5 antibodies. As shown in Fig. 1B, ApoA5 was pulled down with the anti-ANGPTL3 antibody and with the anti-ANGPTL8 antibody. In addition, this figure also shows that the anti-ANGPTL3 antibody pulled down ANGPTL8 (indicating that the anti-ANGPTL3 antibody binds the ANGPTL3/8 complex) and that the anti-ANGPTL8 antibody pulled down ANGPTL3 (indicating that the anti-ANGPTL8 antibody also binds the ANGPTL3/8 complex). These data thus supported the concept that ApoA5 detected when pull downs were performed with anti-ANGPTL3 and anti-ANGPTL8 antibodies were due to ApoA5 being associated with the ANGPTL3/8 complex.

**Fig. 2:**
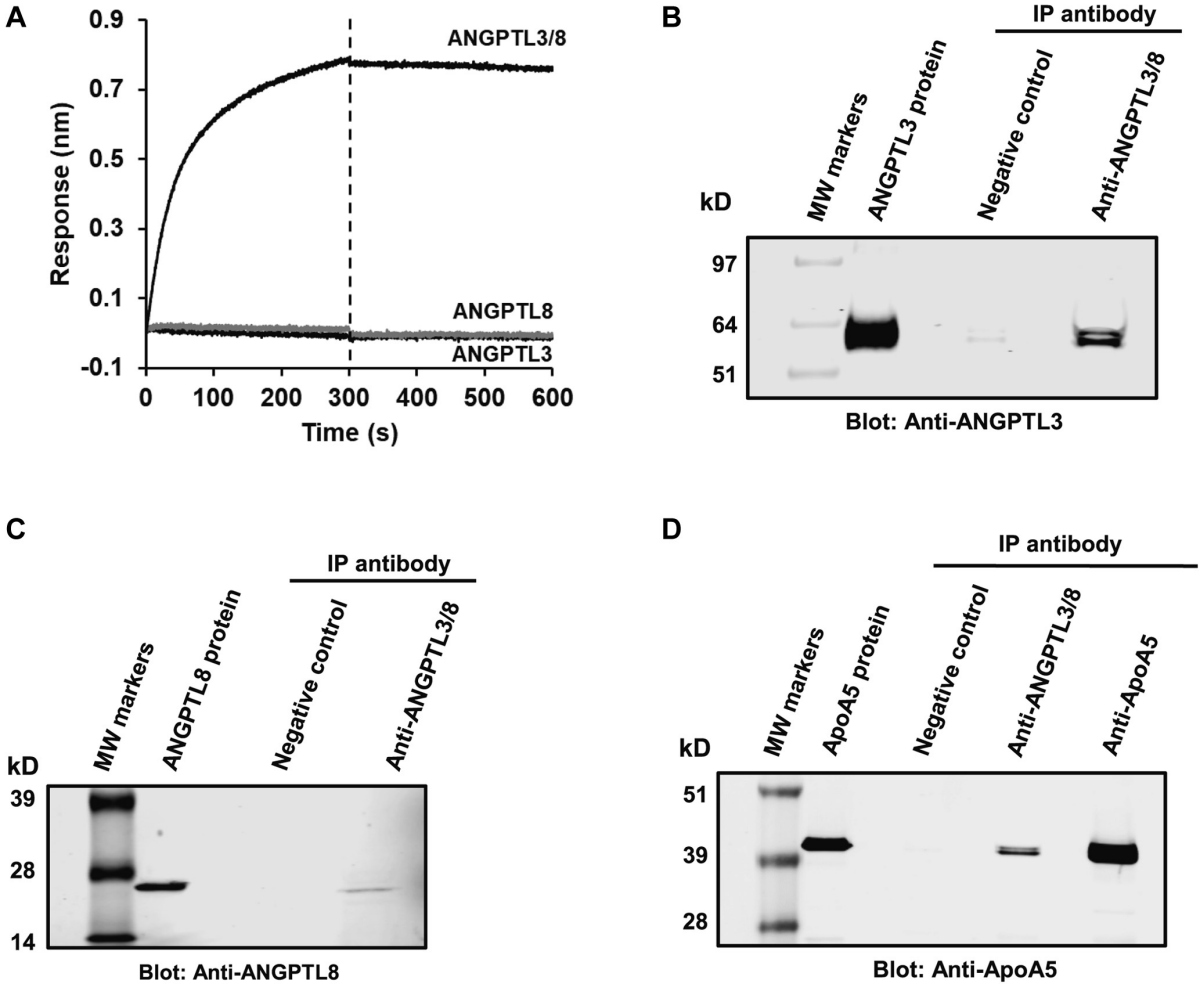
An anti-ANGPTL3/8-specific antibody immunoprecipitates ApoA5 from human serum. A: Biolayer interferometry confirmed the specific binding of the anti-ANGPTL3/8 antibody to ANGPTL3/8 and not free ANGPTL3 or ANGPTL8. B: The anti-ANGPTL3/8 antibody was used to immunoprecipitate human serum overnight at 4°C. Immunoprecipitated proteins were separated, transferred to PVDF, and stained with anti-ANGPTL3 antibody to confirm pull down of ANGPTL3. C: The anti-ANGPTL3/8-specific antibody was used to immunoprecipitate human serum overnight at 4°C. Immunoprecipitated proteins were separated, transferred to PVDF, and stained with anti-ANGPTL8 antibody to confirm pull down of ANGPTL8. D: An irrelevant, negative control antibody, the anti-ANGPTL3/8 antibody, and an anti-ApoA5 antibody were used to immunoprecipitate human serum overnight at 4°C. Immunoprecipitated proteins were separated, transferred to PVDF, and stained with the anti-ApoA5 antibody. Results are representative of two independent experiments. ANGPTL8, angiopoietin-like protein 8.

To confirm further the association of ApoA5 with the ANGPTL3/8 complex, we developed an anti-ANGPTL3/8 complex–specific antibody and utilized biolayer interferometry to demonstrate its specificity for ANGPTL3/8 versus ANGPTL3 or ANGPTL8 alone. Figure 2A shows the biolayer interferometry results, in which the anti-ANGPTL3/8 antibody recognized only the ANGPTL3/8 complex and not free ANGPTL3 or ANGPTL8. We also confirmed the ability of the anti-ANGPTL3/8 antibody to bind ANGPTL3/8 complex in human serum by immunoprecipitating human serum with the antibody and performing Western blotting with anti-ANGPTL3 or anti-ANGPTL8 antibodies. As shown in Fig. 2B and C, the anti-ANGPTL3/8 antibody pulled down both ANGPTL3 and ANGPTL8 from human serum, indicating that it was capable of immunoprecipitating ANGPTL3/8 complex present in human serum. We next immunoprecipitated human serum with the anti-ANGPTL3/8 antibody, an anti-ApoA5 antibody, and an irrelevant negative control antibody and performed Western blotting with an anti-ApoA5 antibody. As Fig. 2D shows, ApoA5 coimmunoprecipitated with ANGPTL3/8, thus confirming the association of ApoA5 with the ANGPTL3/8 complex in human serum that was observed via MS.

We also utilized an MS LC-MRM method with SIL peptides to ascertain the protein ratio of ApoA5 to ANGPTL3/8 in the ApoA5/ANGPTL3/8 complex present in human serum. In these experiments, an anti-ANGPTL3/8 antibody was used to immunoprecipitate human serum, and immunoprecipitated proteins were enzymatically digested. Chromatograms of MRM peptide ions detected in the anti-ANGPTL3/8 immunoprecipitation samples are shown in Supplemental Fig. S18–S20, and the ANGPTL3/8 and ApoA5 MRM peptide characteristics are shown in Supplemental Table S2. By using identical molar amounts of SIL ANGPTL3, ANGPTL8, and ApoA5 peptides spiked into the samples during the digests, we were able to confirm that the ratio of ANGPTL3 to ANGPTL8 was 3:1 (consistent with our previous observations for the ANGPTL3/8 complex) (11) and that the ratio of ApoA5 to ANGPTL8 was 0.5:1 (Table 1). Because the ANGPTL3/8 complex contains one molecule of ANGPTL8, the ratio of ApoA5 to ANGPTL3/8 in the ApoA5/ANGPTL3/8 complex was therefore also determined to be 0.5:1, suggesting that ApoA5 is bound to roughly half of the ANGPTL3/8 complexes in human serum.

**Table 1:** Determination of the ApoA5/ANGPTL3/8 complex protein ratio by MS

Sample		ANGPTL3 (pmole)			ANGPTL8 (pmole)			ApoA5 (pmole)			Protein Ratios	
		Peptide #1	Peptide #2	Average	Peptide #1	Peptide #2	Average	Peptide #1	Peptide #2	Average	ANGPTL3/8	ApoA5/ANGPTL8
	Sample #1	0.133	0.150	0.141	0.048	0.054	0.051	0.028	0.027	0.027	2.8	0.53
	Sample #2	0.136	0.155	0.145	0.050	0.054	0.052	0.025	0.027	0.026	2.8	0.50
	Sample #3	0.130	0.152	0.141	0.052	0.053	0.052	0.025	0.028	0.026	2.7	0.50
	SD	0.003	0.003	0.002	0.002	0.001	0.001	0.002	0.001	0.001	0.06	0.02

ANGPTL8, angiopoietin-like protein

An anti-ANGPTL3/8 antibody was used to immunoprecipitate ANGPTL3/8 and any ApoA5 bound to it from three different human serum pools (1 ml each). Immunoprecipitated proteins were digested using trypsin and Lys-C. Identical molar amounts (0.25 pmole) of stable isotope–labeled (SIL) ANGPTL3, ANGPTL8, and ApoA5 peptides were spiked into samples during digestion, and the ratios of unlabeled-to-labeled peptides were determined using LC-MRM. The molar amounts of endogenous peptides in each sample were then calculated. Stoichiometries of protein complexes were determined by comparing the averaged ratios derived from two peptides per protein. SDs are shown for each peptide in the technical replicates and for the overall protein ratios. Because the ratio of ANGPTL3 to ANGPTL8 in the ANGPTL3/8 complex was 3:1 and the ratio of ApoA5 to ANGPTL8 was 0.5:1, the ratio of ApoA5 to ANGPTL3/8 in the ApoA5/ANGPTL3/8 complex was determined to be 0.5:1. These results thus suggest that approximately half of circulating ANGPTL3/8 complexes are bound to ApoA5.

### Generation of recombinant ApoA5 protein

**Fig. 3:**
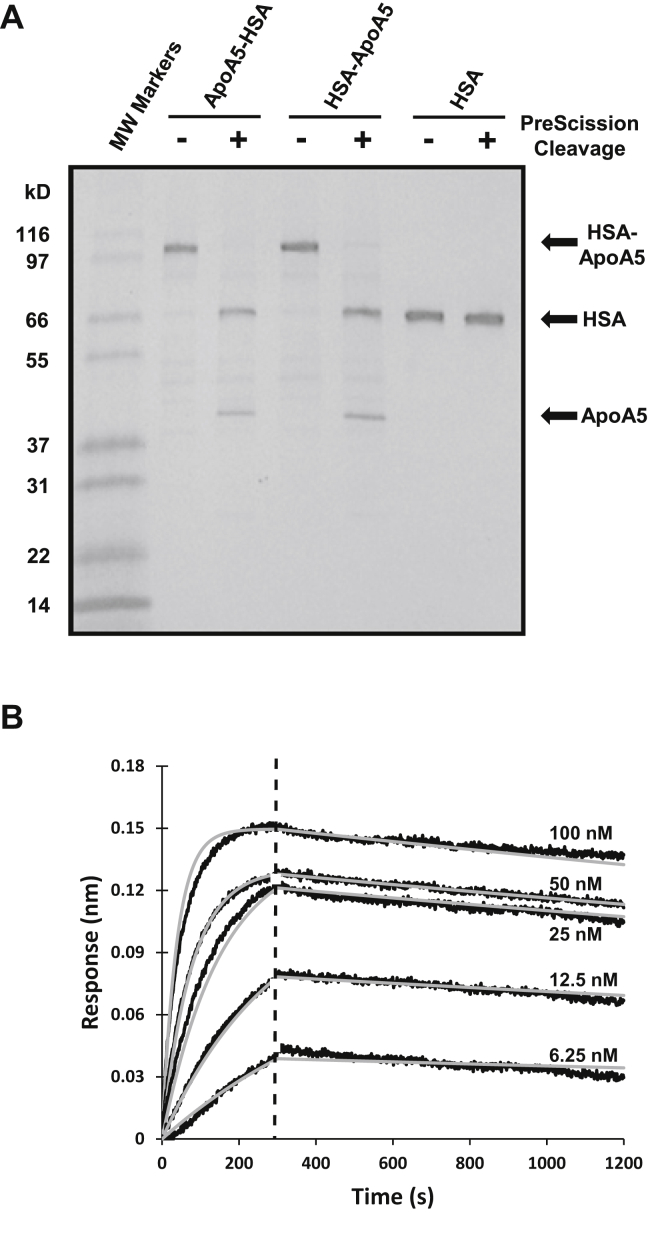
Expression and purification of recombinant ApoA5 protein and characterization of its binding to the ANGPTL3/8 complex. A: Recombinant human ApoA5-HSA, HSA-ApoA5, and control HSA protein (0.5 μg of each) were analyzed either without or with PreScission cleavage of the HSA tag. Proteins were separated via gradient gel electrophoresis using a 4%–20% Tris-glycine gel and stained with Coomassie Blue. B: Biolayer interferometry was used to examine the binding affinity of ApoA5 to the ANGPTL3/8 complex. Flag-tagged ANGPTL3/8 complex was first immobilized on streptavidin biosensors coated with anti-Flag antibody. Immobilized ANGPTL3/8 was then incubated with increasing concentrations of ApoA5 (6.25–100 nM) to monitor association of ApoA5 with the ANGPTL3/8 complex and then transferred into buffer-only wells to monitor its dissociation from the ANGPTL3/8 complex, with background reference subtraction performed. ApoA5 binding to ANGPTL3/8 demonstrated a moderate on-rate (association) and a very slow off-rate (dissociation). Curves were fit globally using a 1:1 binding model to generate k_on_, k_off_, and K_D_ values of 2.60 × 10^5^ M^−1^s^−1^, 1.35 × 10^−4^ s^−1^, and 520 pM, respectively. Raw data were plotted in black and the curve fits in gray. Results are representative of three independent experiments. ANGPTL8, angiopoietin-like protein 8; HSA, human serum albumin.

Initial attempts to express human ApoA5 were unsuccessful, with the recombinant protein proving to be unstable. We considered reports such as those by Castleberry *et al*. (36) and opted to take a new approach by performing mammalian expression of ApoA5 coupled to His tag-mature HSA at either the N terminus (HSA-ApoA5) or the C terminus (ApoA5-HSA) of ApoA5. The ApoA5 could then be used either as an intact ApoA5 fusion protein or could undergo PreScission cleavage to generate ApoA5 (and HSA) immediately before use. Figure 3A shows a Coomassie-stained gel, with ApoA5-HSA, HSA-ApoA5, and HSA alone evaluated either without or with PreScission cleavage. As the figure demonstrates, this approach provided ApoA5 suitable for subsequent experiments.

To characterize the recombinant ApoA5 protein more fully and confirm its ability to bind to recombinant ANGPTL3/8, we examined the binding of ApoA5 to ANGPTL3/8, using biolayer interferometry. In these experiments, shown in Fig. 3B, ANGPTL3/8 was immobilized on the solid-phase biosensor, and its binding to the solution phase ApoA5 was measured via surface plasmon resonance. Curve fitting performed with a 1:1 binding model estimated an on-rate (k_on_) of 2.60 × 10^5^ M^−1^s^−1^, an off-rate (k_off_) of 1.35 × 10^−4^ s^−1^, and an equilibrium dissociation constant (K_D_) of 520 pM, thus indicating an extremely slow dissociation for the binding. These binding characteristics were consistent with our ability to coimmunoprecipitate ApoA5 with ANGPTL3/8 from human serum and indicated that our recombinant ApoA5 should be suitable for LPL functional activity experiments.

### ApoA5 suppression of ANGPTL3/8-mediated LPL-inhibitory activity

**Fig. 4:**
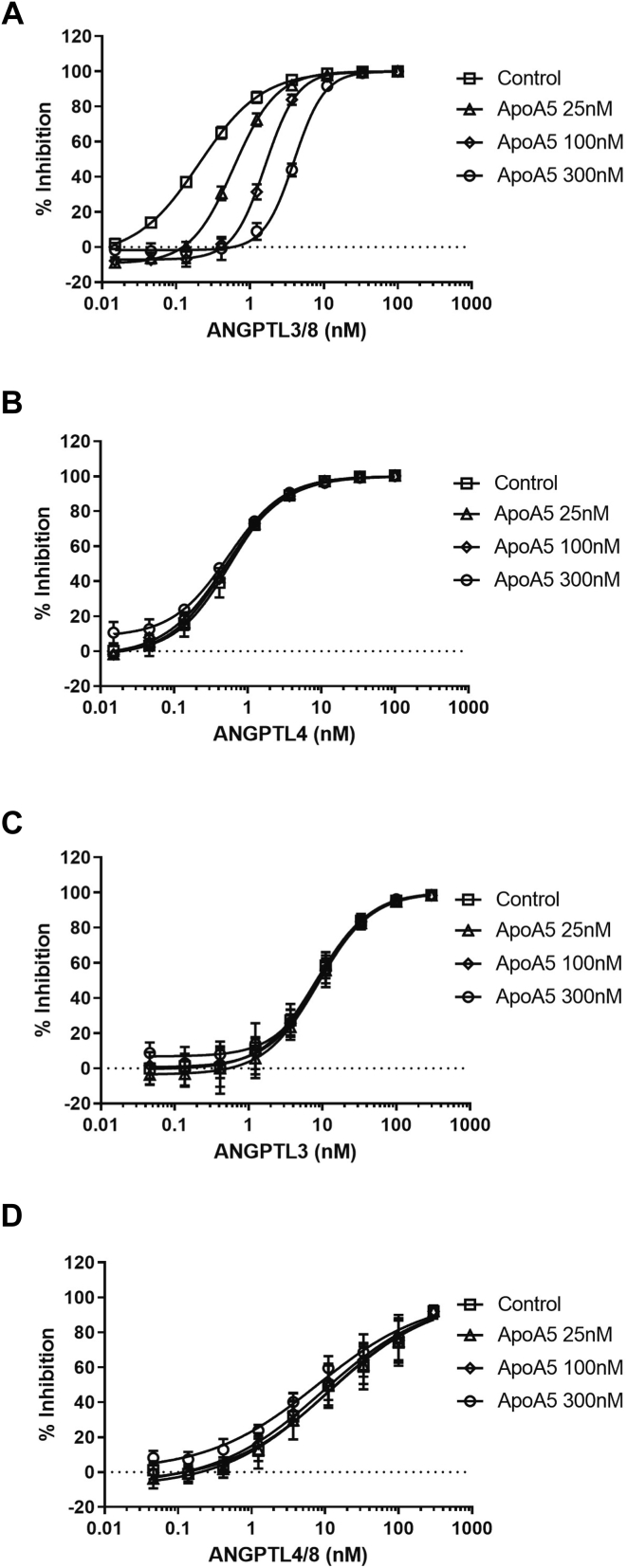
ApoA5 selectively blocks the ability of ANGPTL3/8 to inhibit LPL activity. The ability of ANGPTL3/8, ANGPTL4, ANGPTL3, and ANGPTL4/8 to inhibit the LPL activity in the presence of 0 nM (squares), 25 nM (triangles), 100 nM (diamonds), and 300 nM (circles) of ApoA5 (HSA-ApoA5) was assessed using LPL-stable expression cells with EnzChek fluorescent lipase substrate. All results are shown as the mean ± SD (n = 3 from three independent experiments). A: ANGPTL3/8 was preincubated with ApoA5 before the addition of lipase substrate. B: ANGPTL4 was preincubated with ApoA5 before the addition of lipase substrate. C: ANGPTL3 was preincubated with ApoA5 before the addition of lipase substrate. D: ANGPTL4/8 was preincubated with ApoA5 before the addition of lipase substrate. ANGPTL8, angiopoietin-like protein 8; HSA, human serum albumin.

Upon observing the association of ApoA5 with ANGPTL3/8 in human serum, we sought to determine if ApoA5 altered the ability of ANGPTL3/8 to inhibit the LPL activity. To do this, we assessed the ability of increasing concentrations of ANGPTL3/8 to inhibit the LPL activity in the presence of 0–300 nM ApoA5 using EnzChek fluorescent lipase substrate. Figure 4A shows the results of these experiments, in which HSA-ApoA5 dose-dependently decreased the ability of ANGPTL3/8 to inhibit the LPL activity. In contrast, when similar experiments were performed with ANGPTL4 (which inhibits LPL activity to roughly the same degree as ANGPTL3/8), there was no decrease observed in the ability of ANGPTL4 to inhibit LPL in the presence of increasing concentrations of ApoA5 (Fig. 4B). Similarly, when the same experiments were performed with ANGPTL3 and ANGPTL4/8 (which are both relatively weak inhibitors of LPL), there was no ApoA5-mediated decrease in their ability to inhibit LPL (Fig. 4C and D, respectively).

**Fig. 5:**
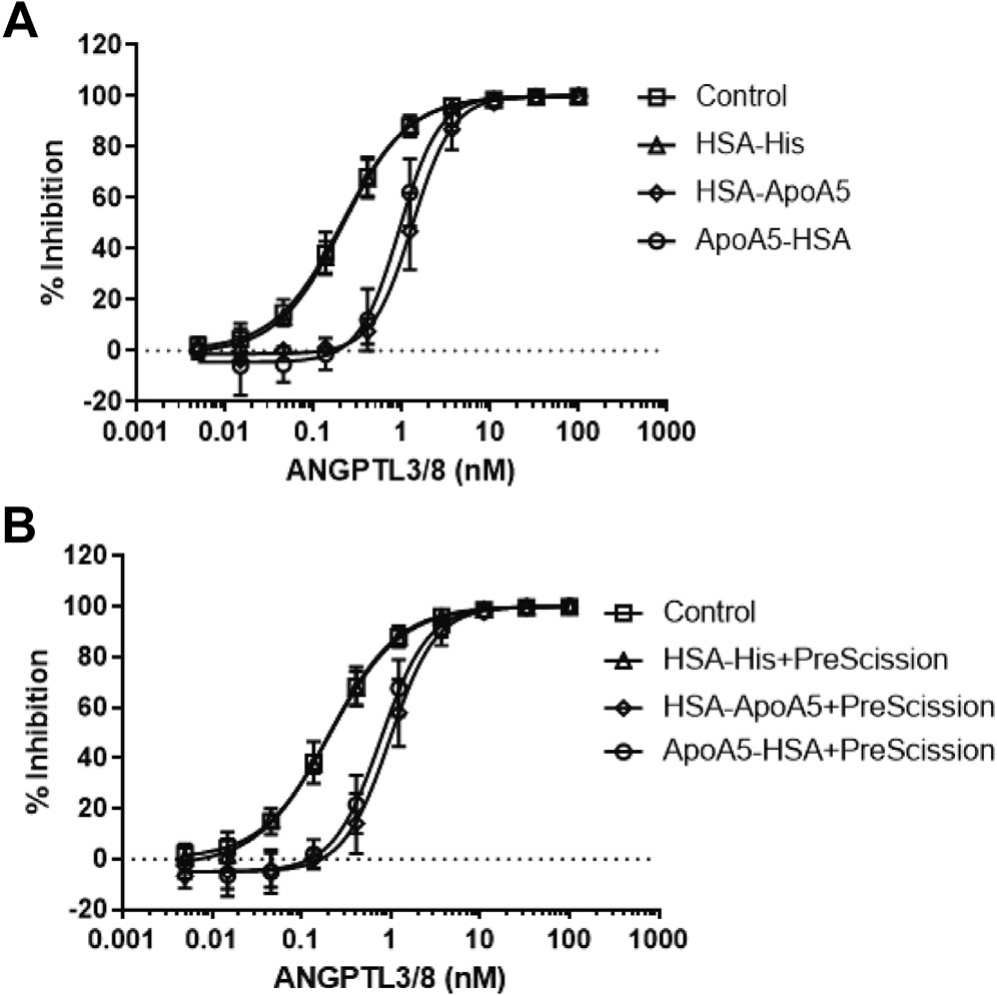
HSA-ApoA5 and ApoA5-HSA block ANGPTL3/8-mediated LPL inhibitory activity in a similar manner to PreScission-cleaved HSA-ApoA5 and PreScission-cleaved ApoA5-HSA. A: The ability of 100 nM HSA-ApoA5 (diamonds) and 100 nM ApoA5-HSA (circles) to inhibit LPL activity in the presence of increasing concentrations of ANGPTL3/8 was assessed using LPL-stable expression cells with EnzChek fluorescent lipase substrate. Control (squares) and HSA-His–alone (triangles) conditions are also shown. Results are shown as the mean ± SD (n = 3 from three independent experiments). B: The ability of 100 nM PreScission-cleaved HSA-ApoA5 (diamonds) and 100 nM PreScission-cleaved ApoA5-HSA (circles) to inhibit the LPL activity in the presence of increasing concentrations of ANGPTL3/8 was assessed using LPL-stable expression cells with EnzChek fluorescent lipase substrate. Control (squares) and HSA-His alone plus PreScission cleavage (triangles) are also shown. Results are shown as the mean ± SD (n = 3 from three independent experiments). ANGPTL8, angiopoietin-like protein 8; HSA, human serum albumin.

Comparable results were also obtained when using ApoA5-HSA, PreScission-cleaved HSA-ApoA5, or PreScission-cleaved ApoA5-HSA. As Fig. 5A shows, HSA-ApoA5 and ApoA5-HSA demonstrated similar suppression of ANGPTL3/8-mediated LPL-inhibitory activity. Likewise, as shown in Fig. 5B, PreScission-cleaved HSA-ApoA5 and PreScission-cleaved ApoA5-HSA also demonstrated comparable suppression of ANGPTL3/8-mediated LPL-inhibitory activity while HSA itself had no effect on ANGPTL3/8-mediated LPL-inhibitory activity. Together, these data confirmed the results in Fig. 4 and led us to conduct future LPL activity assays with HSA-ApoA5.

**Fig. 6:**
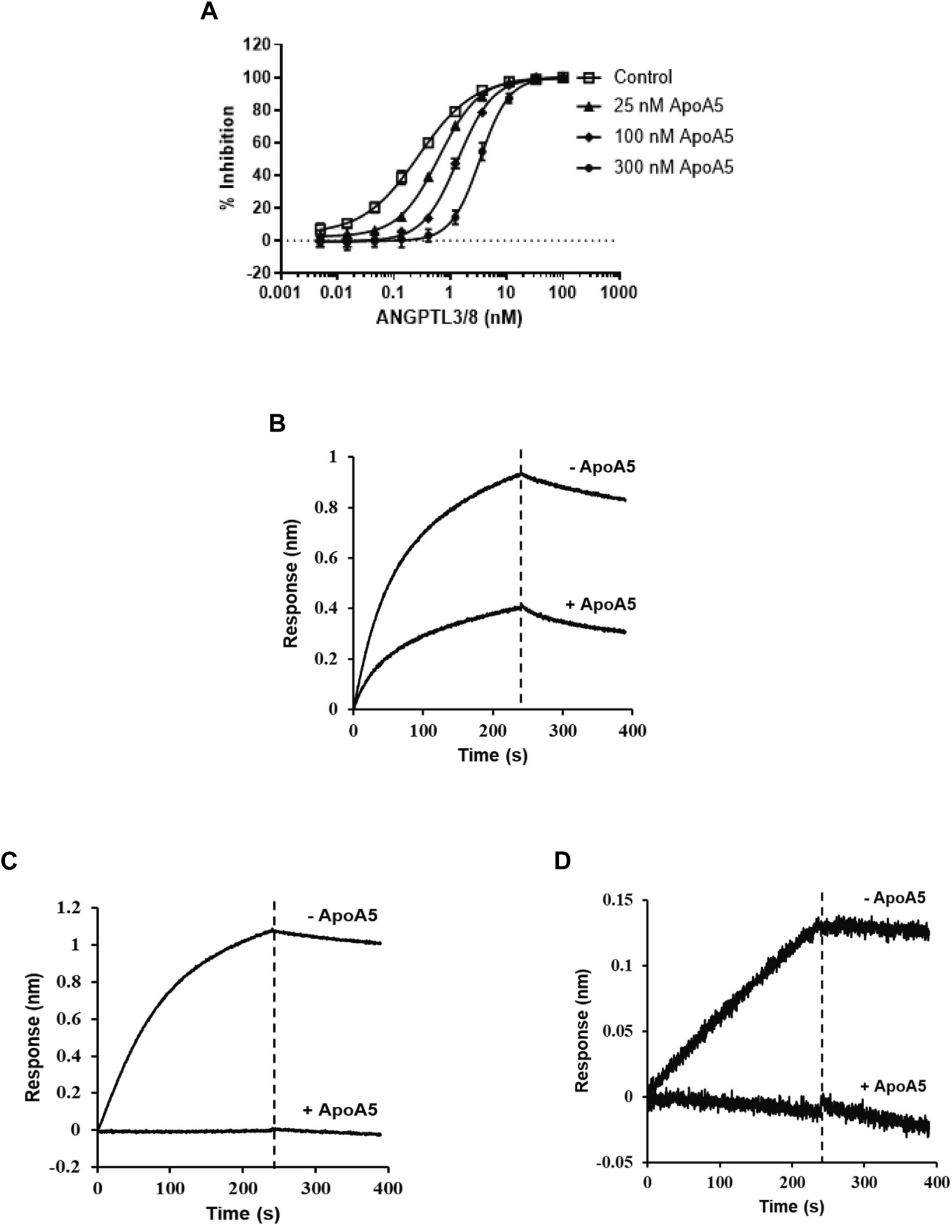
Further characterization of ApoA5 suppression of ANGPTL3/8-mediated LPL inhibition. A: The ability of ANGPTL3/8 to inhibit LPL activity using VLDL as a substrate was assessed in the presence of 0 nM (squares), 25 nM (triangles), 100 nM (diamonds), and 300 nM (circles) of ApoA5 (HSA-ApoA5). The assay was performed with VLDL as a substrate, and NEFA levels were measured. Results are shown as the mean ± SD (n = 4 from two independent experiments). B: Biolayer interferometry was used to assess the binding of the immobilized anti-ANGPTL3/8 antibody to the ANGPTL3/8 complex after preincubation of ANGPTL3/8 with ApoA5. (C) Biolayer interferometry was used to assess the binding of the immobilized anti-ANGPTL3 antibody to the ANGPTL3/8 complex after preincubation of ANGPTL3/8 with ApoA5. D: Biolayer interferometry was used to assess the binding of the immobilized anti-ANGPTL8 antibody to the ANGPTL3/8 complex after preincubation of ANGPTL3/8 with ApoA5. ANGPTL8, angiopoietin-like protein 8.

Because our LPL functional assays were conducted with EnzChek fluorescent lipase substrate, we also sought to confirm the ability of ApoA5 to suppress the LPL-inhibitory activity of ANGPTL3/8 in an assay in which LPL activity could be quantitated by measuring the release of NEFA from the more natural substrate VLDL. To do this, we repeated the previous LPL activity experiments examining the ability of ApoA5 to suppress ANGPTL3/8-mediated inhibition of LPL, but with VLDL as a substrate to measure the release of NEFA. As Fig. 6A shows, very similar results were obtained when VLDL was used as an LPL substrate, thus confirming the ability of ApoA5 to block ANGPTL3/8-mediated inhibition of LPL when a more physiological LPL substrate was used.

To understand better the mechanism by which ApoA5 blocked the ability of ANGPTL3/8 to inhibit the LPL activity, we used biolayer interferometry to assess the binding of anti-ANGPTL3, anti-ANGPTL8, and anti-ANGPTL3/8 antibodies to ANGPTL3/8 after preincubation of the ANGPTL3/8 complex with ApoA5. As shown in Fig. 6B, preincubation of ApoA5 with ANGPTL3/8 partially blocked the binding of the anti-ANGPTL3/8 antibody. In comparison, as shown in Fig. 6C and D, the preincubation of ApoA5 with ANGPTL3/8 completely blocked the binding of the anti-ANGPTL3 and anti-ANGPTL8 antibodies, suggesting that little or no-free ANGPTL3 or ANGPTL8 were present after preincubation of ANGPTL3/8 with ApoA5. Although not completely definitive, the overall results from these experiments suggested that ApoA5 may not act to disrupt the ANGPTL3/8 complex but rather may act to block the ability of the ANGPTL3/8 complex to bind its target.

### Kinetic analyses

**Fig. 7:**
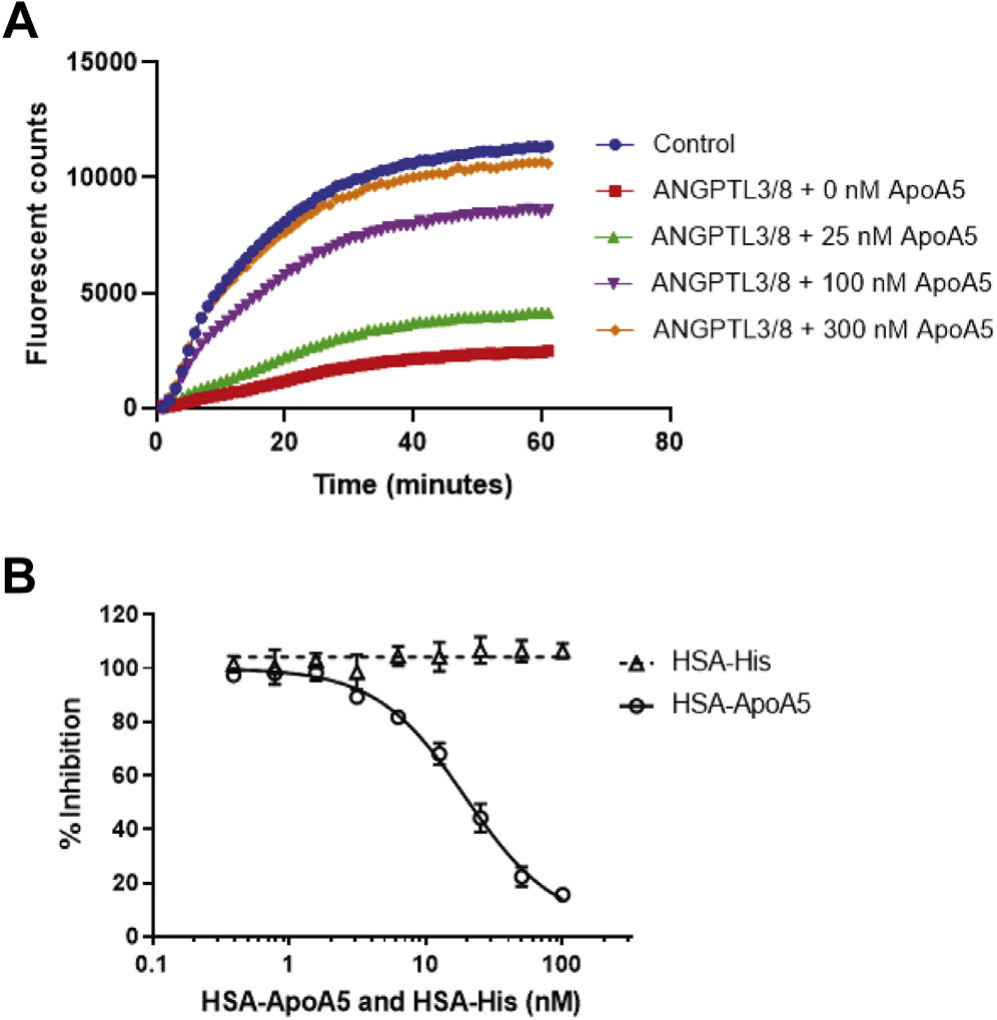
Analyses of ApoA5 effect on ANGPTL3/8-mediated LPL inhibition. A: The ability of 1.2 nM of ANGPTL3/8 to inhibit LPL activity in the presence of HSA-ApoA5 was assessed via kinetic analyses using LPL-stable expression cells with EnzChek fluorescent lipase substrate. ANGPTL3/8 was preincubated with 0 nM (red squares), 25 nM (green triangles), 100 nM (purple triangles), or 300 nM (orange diamonds) of HSA-ApoA5 before the addition of lipase substrate. The control condition (blue circles) indicates the absence of ANGPTL3/8 and ApoA5. Results are representative of three independent experiments. B: The effect of HSA-ApoA5 (circles) on the ability of 0.3 nM ANGPTL3/8 (the IC_60_ of ANGPTL3/8) to inhibit LPL was assessed using LPL-stable expression cells. ANGPTL3/8 was preincubated with increasing concentrations of HSA-ApoA5 before the addition of lipase substrate. The effect of HSA alone (triangles) was also evaluated. Results are shown as the mean ± SD (n = 4 from two independent experiments). ANGPTL8, angiopoietin-like protein 8; HSA, human serum albumin.

To study in more detail the effect of ApoA5 on ANGPTL3/8, we performed kinetic analyses of the LPL-inhibitory activity of 1.2 nM of the ANGPTL3/8 complex in the presence of increasing concentrations of ApoA5. As Fig. 7A shows, at a concentration of 25 nM ApoA5, a decrease in the ability of ANGPTL3/8 to inhibit the LPL activity was clearly evident. At 100 nM of ApoA5, the ability of ANGPTL3/8 to inhibit the LPL activity was decreased by more than two-thirds. At 300 nM of ApoA5, the ability of ANGPTL3/8 to inhibit the LPL activity was almost completely blocked. The effect of ApoA5 was thus more than half-maximal at concentrations of 100 nM ApoA5 and 1.2 nM of ANGPTL3/8, where the molar ratio was 83:1.

To probe this concept further, we directly assessed the effect of increasing concentrations of ApoA5 on the ability of 0.3 nM ANGPTL3/8 (the approximate IC_60_ for ANGPTL3/8) to inhibit LPL. In these experiments, 0.3 nM of ANGPTL3/8 was first preincubated with increasing concentrations of ApoA5 before its evaluation in the LPL assay. As Fig. 7B demonstrates, the EC_50_ for ApoA5 to block 0.3 nM ANGPTL3/8-mediated inhibition of the LPL activity was 21 nM, for a molar ratio of ApoA5:ANGPTL3/8 of 70:1. This molar ratio was very close to the 83:1 ratio estimated from the kinetic analyses.

**Fig. 8:**
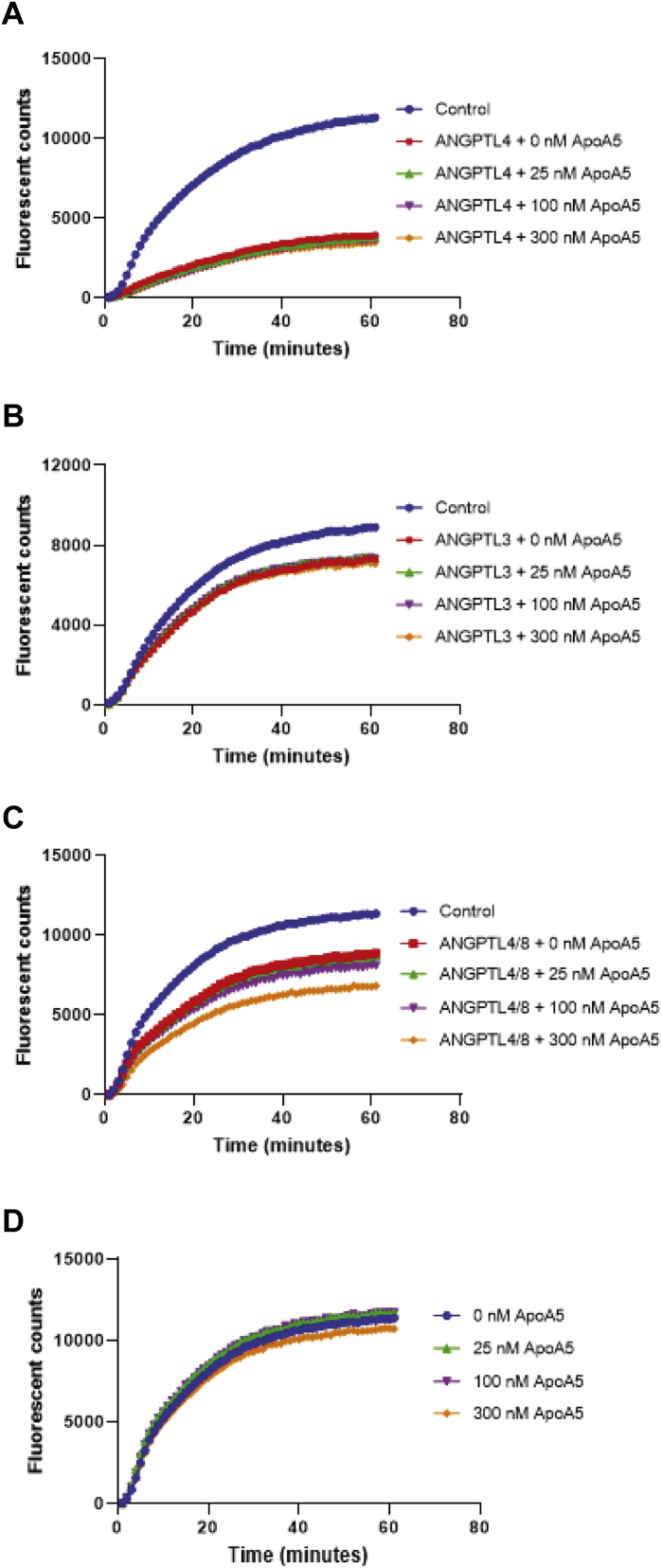
Kinetic analyses of the effect of ApoA5 on LPL-inhibitory activities of ANGPTL4, ANGPTL3, and ANGPTL4/8. The ability of ANGPTL4, ANGPTL3, or ANGPTL4/8 (each at 1.2 nM) to inhibit LPL activity in the presence of different concentrations of ApoA5 (HSA-ApoA5) at 0 nM (red squares), 25 nM (green triangles), 100 nM (purple triangles), and 300 nM (orange diamonds) was assessed using LPL-stable expression cells with EnzChek fluorescent lipase substrate. The control condition (blue circles) indicates the absence of ApoA5 and each respective ANGPTL protein or complex. All results are representative of three independent experiments. (A) ANGPTL4 was preincubated with ApoA5 before the addition of lipase substrate. B: ANGPTL3 was preincubated with ApoA5 before the addition of lipase substrate. C: ANGPTL4/8 was preincubated with ApoA5 before the addition of lipase substrate. D: The effect of ApoA5 alone at 0 nM (blue circles), 25 nM (green triangles), 100 nM (purple triangles), and 300 nM (orange diamonds) on the LPL activity was assessed using LPL-stable expression cells. ANGPTL8, angiopoietin-like protein 8; HSA, human serum albumin.

To confirm further that the observed effect of ApoA5 to suppress the LPL-inhibitory activity of ANGPTL3/8 was specific for ANGPTL3/8, we performed additional kinetic analyses of the LPL-inhibitory activities of 1.2 nM of ANGPTL4, ANGPTL3, and ANGPTL4/8 complex in the presence of increasing concentrations of ApoA5. As Fig. 8A–C demonstrate, ApoA5 did not suppress the ability of ANGPTL4, ANGPTL3, or ANGPTL4/8 to inhibit the LPL activity (in the case of ANGPTL4/8, there was a very slight trend toward increasing the LPL-inhibitory activity). These experiments thus verified that the ability of ApoA5 to suppress the LPL-inhibitory activity of the ANGPTL3/8 complex was specific for ANGPTL3/8 and that this property was not shared with regard to any of the other ANGPTL proteins or complexes tested. Importantly, as Fig. 8D shows, there was no ability of ApoA5 alone to stimulate LPL activity, indicating that ApoA5 on its own was not capable of directly increasing LPL activity but rather could only act indirectly to increase LPL activity by decreasing the ability of ANGPTL3/8 to inhibit LPL.

### Human ApoA5 suppression of mouse ANGPTL3/8-mediated inhibition of mouse LPL activity

After obtaining the above results, we contemplated previous reports of the in vivo effect of human ApoA5 in mice. As shown by Nelbach *et al*. (37), overexpression of human ApoA5 in mice lacking endogenous ApoA5 resulted in TG lowering; however, the mice had average circulating human ApoA5 levels of roughly 12.5 μg/ml, which are approximately 50–100 times higher than actual circulating human concentrations. Similarly, to see an effect of human ApoA5 protein in mice lacking endogenous ApoA5, Shu *et al*. (38) elegantly injected the mice with large quantities of reconstituted human ApoA5-HDL to achieve circulating human ApoA5 levels that were again roughly 50–100 times higher than actual human serum levels to demonstrate TG lowering.

**Fig. 9:**
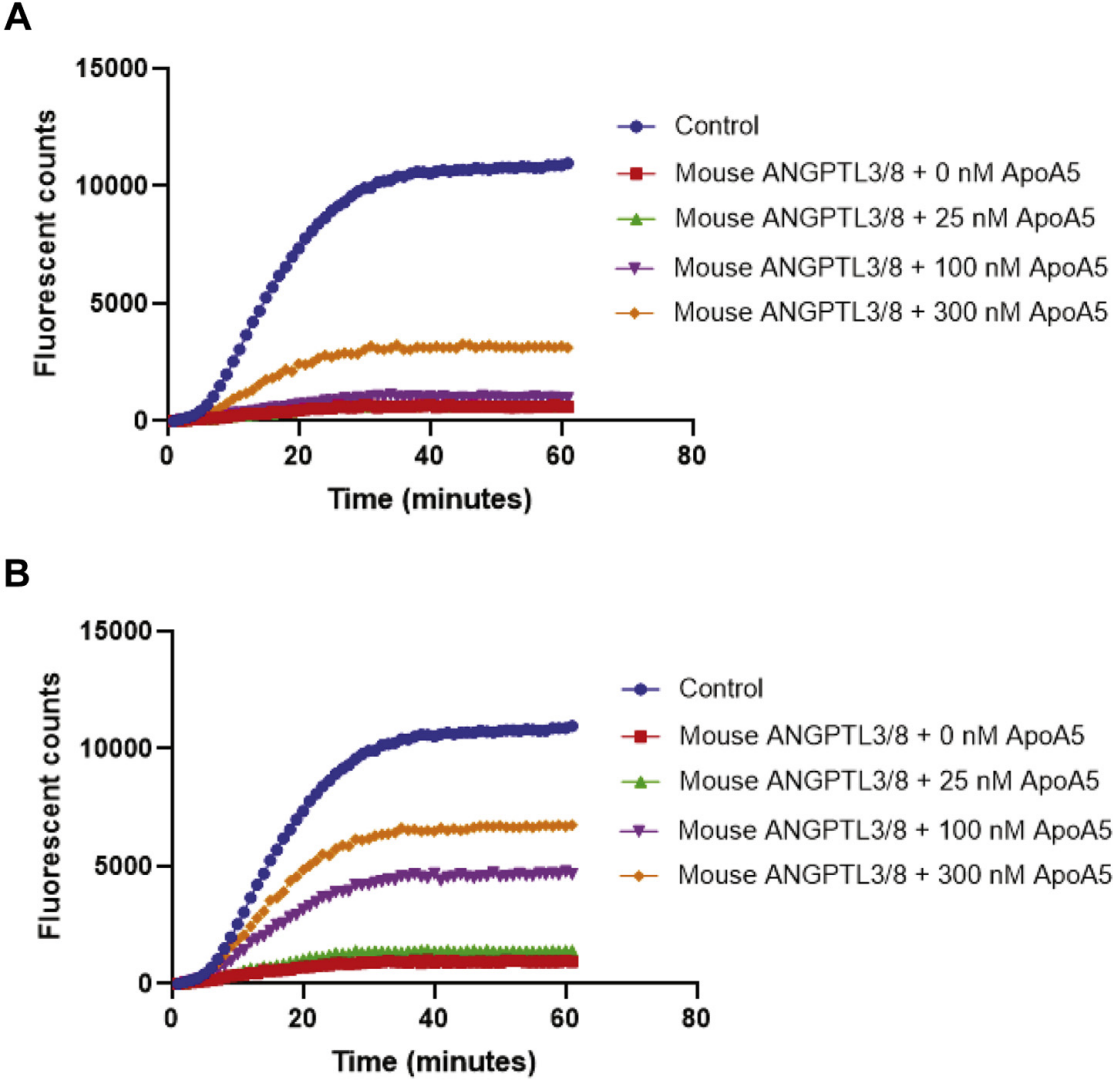
Human ApoA5 shows markedly decreased potency in suppressing mouse ANGPTL3/8-mediated inhibition of mouse LPL activity. A: Kinetic analyses were performed to assess the effect of human ApoA5 on mouse ANGPTL3/8. The ability of 1.2 nM mouse ANGPTL3/8 to inhibit mouse LPL in the presence of different concentrations of human ApoA5 (HSA-ApoA5) at 0 nM (red squares), 25 nM (green triangles), 100 nM (purple triangles), and 300 nM (orange diamonds) was assessed using mouse LPL-stable expression cells with EnzChek fluorescent lipase substrate. The control condition (blue circles) indicates the absence of both human ApoA5 and mouse ANGPTL3/8. Results are representative of three independent experiments. B: The ability of 0.4 nM mouse ANGPTL3/8 to inhibit mouse LPL activity in the presence of the same concentrations of human ApoA5 used in Fig. 9A was assessed using mouse LPL-stable expression cells with EnzChek fluorescent lipase substrate. The control condition indicates the absence of both human ApoA5 and mouse ANGPTL3/8. Results are representative of 3 independent experiments. ANGPTL8, angiopoietin-like protein 8.

These findings suggested to us that human ApoA5 may not be nearly as potent in suppressing mouse ANGPTL3/8-medated inhibition of mouse LPL activity as it is in suppressing human ANGPTL3/8-mediated inhibition of human LPL activity. To test this hypothesis, we performed kinetic analyses of the mouse LPL-inhibitory activity of 1.2 nM mouse ANGPTL3/8 complex in the presence of increasing concentrations of human ApoA5. As Fig. 9A shows, human ApoA5 had a dramatically reduced ability to suppress the inhibition of mouse LPL activity caused by 1.2 nM mouse ANGPTL3/8. At 25 and 100 nM of human ApoA5, there was virtually no effect on 1.2 nM mouse ANGPTL3/8-mediated inhibition of mouse LPL activity. Even at 300 nM of human ApoA5, the suppression of 1.2 nM mouse ANGPTL3/8-mediated LPL inhibition was relatively minimal. These results obtained regarding human ApoA5 suppression of 1.2 nM mouse ANGPTL3/8-mediated inhibition of mouse LPL activity stand in stark contrast to the ability of human ApoA5 to suppress 1.2 nM human ANGPTL3/8-mediated inhibition of human LPL activity that was previously observed in Fig. 7A.

To explore further this difference with regard to the decreased potency of human ApoA5 against mouse ANGPTL3/8 versus human ANGPTL3/8, we performed the same experiment described above, but with a decreased concentration of 0.4 nM mouse ANGPTL3/8 complex. As Fig. 9B shows, human ApoA5 was not nearly as effective in suppressing the LPL inhibitory activity of even 0.4 nM mouse ANGPTL3/8 as it was in suppressing the LPL inhibitory activity of 1.2 nM human ANGPTL3/8 (a 3-fold greater concentration of the corresponding ANGPTL3/8 complex). Together, these results demonstrated that human ApoA5 had markedly reduced potency against mouse ANGPTL3/8 versus human ANGPTL3/8 and thus help explain why supraphysiological levels of human ApoA5 have been required to lower TG in mice (37, 38).

### Insulin and LXR agonist-stimulated secretion of ANGPTL3/8 and ApoA5 from hepatocytes

In light of the above results showing that ApoA5 selectively suppressed the LPL-inhibitory activity of ANGPTL3/8, we considered previous reports showing that LXR agonists cause hypertriglyceridemia and decrease the expression of ApoA5 (32, 33). We hypothesized that the reported increases in TG levels after administration of LXR agonists might be due more to increased hepatic secretion of ANGPTL3/8 than to decreased ApoA5 secretion. We also considered that LXR agonist–induced increases in hepatic ANGPTL3/8 secretion might be further augmented by insulin because we previously demonstrated that insulin stimulated the secretion of ANGPTL3/8 from hepatocytes (11).

**Fig. 10:**
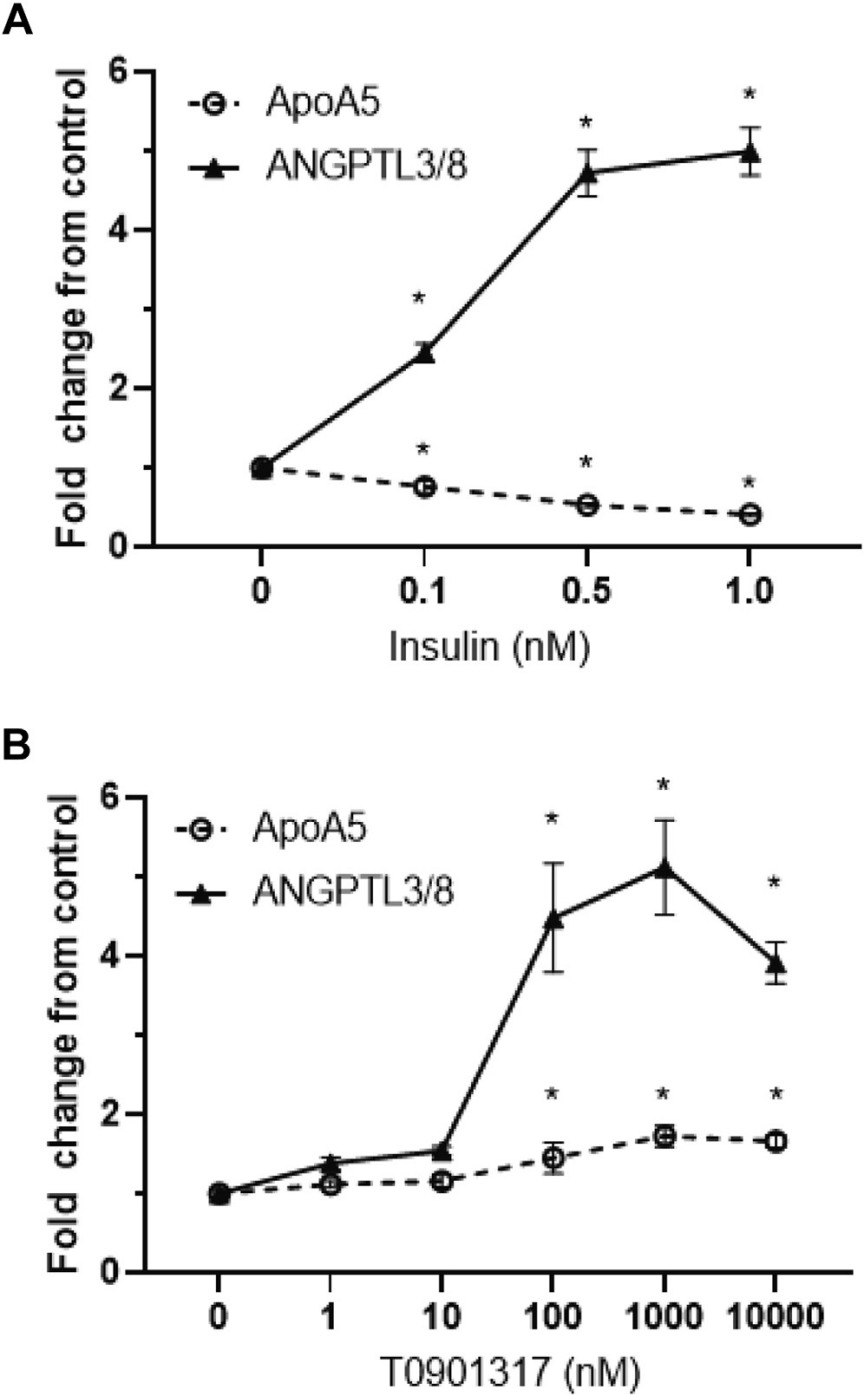
Effect of insulin or the LXR agonist T0901317 on hepatocyte secretion of ANGPTL3/8 and ApoA5. A: Human primary hepatocytes were preincubated in application media in the absence of insulin. After aspiration, cells were incubated with application media in the presence of 0–1 nM insulin. ANGPTL3/8 (filled triangles) and ApoA5 (open circles) secreted into the media were measured using immunoassays, with the results shown as the mean ± SEM (n = 8 from 2 independent experiments, ∗*P* < 0.01). B: Human primary hepatocytes were preincubated in application media in the absence of insulin. After aspiration, cells were incubated with application media in the presence of 0–10,000 nM T0901317. ANGPTL3/8 (filled triangles) and ApoA5 (open circles) secreted into the media were measured using immunoassays, with the results shown as the mean ± SEM (n = 8 from two independent experiments, ∗*P* < 0.01). ANGPTL8, angiopoietin-like protein 8.

To test these hypotheses, we first performed an insulin-response dose curve in primary human hepatocytes and measured secreted ANGPTL3/8 and ApoA5. Figure 10A shows the results from these experiments, in which insulin dose-dependently increased hepatocyte secretion of ANGPTL3/8 while dose-dependently decreasing the secretion of ApoA5. We next performed similar experiments with the LXR agonist T0901317. In these experiments shown in Fig. 10B, T0901317 actually caused a modest dose-dependent increase in ApoA5 secretion but stimulated a marked, dose-dependent increase in ANGPTL3/8 secretion that was far greater in magnitude than the effect observed for ApoA5.

**Fig. 11:**
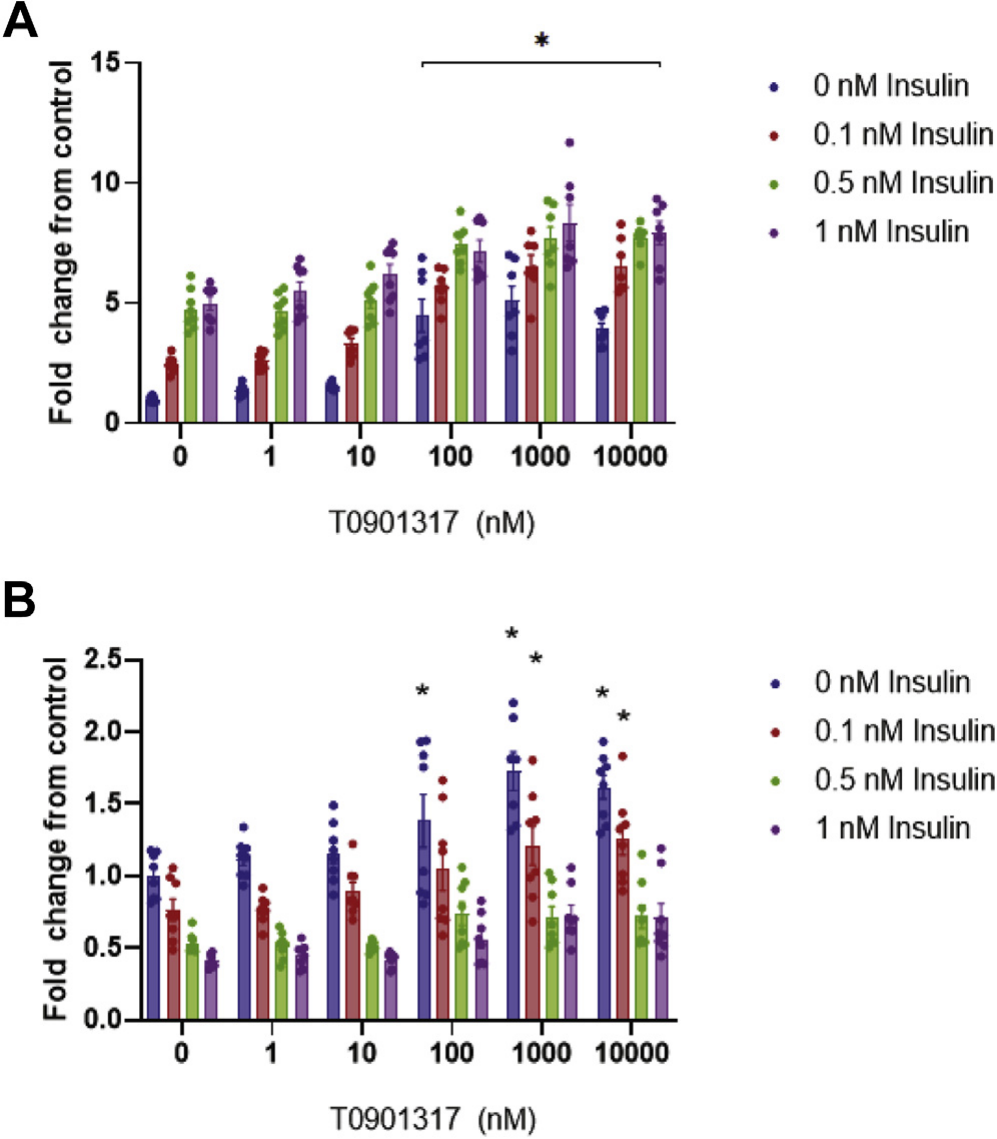
Effect of the combination of insulin and the LXR agonist T0901317 on hepatocyte secretion of ANGPTL3/8 and ApoA5. A: Human primary hepatocytes were preincubated in application media in the absence of insulin. After aspiration, cells were incubated with application media in the presence of 0–1 nM insulin and 0–10,000 nM T0901317. ANGPTL3/8 levels in the media were measured using an immunoassay, with the results shown as the mean ± SEM (n = 8 from two independent experiments, ∗*P* < 0.001). B: ApoA5 levels from the exact same media samples collected in Fig. 11A were measured using an immunoassay, with the results shown as the mean ± SEM (n = 8 from two independent experiments, ∗*P* < 0.01). ANGPTL8, angiopoietin-like protein 8.

After obtaining these results, we next investigated the combined effects of T0901317 and insulin on hepatocyte secretion of ANGPTL3/8 and ApoA5. As Fig. 11A shows, the combination of T0901317 and insulin stimulated ANGPTL3/8 secretion to a greater extent than was seen with either insulin or the LXR agonist alone. When ApoA5 secretion was measured in the same experiments, however, a different pattern emerged. As shown in Fig. 11B, increasing amounts of insulin blocked the ability of T0901317 to stimulate hepatocyte ApoA5 secretion. At 1 nM insulin, ApoA5 secretion was less than that of the control, even in the presence of the maximal concentration of T0901317 tested. Together, these results demonstrated that while T0901317-stimulated ANGPTL3/8 secretion from hepatocytes was enhanced by insulin, T0901317-stimulated ApoA5 secretion from the same hepatocytes was simultaneously attenuated by insulin.

## Discussion

The data in our study reveal a novel intersection of ApoA5 and ANGPTL3/4/8 protein family in the regulation of TG metabolism. Our results indicate that the likely mechanism through which ApoA5 lowers TGs is through suppression of ANGPTL3/8-mediated LPL inhibition. Based on our IP-MS experiments to determine the ratio of ApoA5 to ANGPTL3/8 in ApoA5/ANGPTL3/8 complexes in human serum, we estimate that approximately half of the circulating ANGPTL3/8 complexes have a molecule of ApoA5 bound to them. The very low off-rate that we observed for ApoA5 binding to ANGPTL3/8 suggests the possibility that once a molecule of ApoA5 binds to ANGPTL3/8, ApoA5 inhibition of the ANGPTL3/8 complex might require de novo synthesis of ANGPTL3/8 to overcome the inhibition by ApoA5.

Remarkably, ApoA5 selectively suppressed the LPL-inhibitory activity of the ANGPTL3/8 complex while not decreasing the LPL-inhibitory activity of ANGPTL3, ANGPTL4, or the ANGPTL4/8 complex. Importantly, half-maximal ApoA5 suppression of ANGPTL3/8-mediated LPL-inhibitory activity occurred at an ApoA5:ANGPTL3/8 molar ratio that was consistent with the ApoA5:ANGPTL3/8 molar ratio observed in human serum (11, 30). It should be noted, however, that our in vitro molar ratio of ApoA5:ANGPTL3/8 was obtained using different absolute concentrations of ApoA5 and ANGPTL3/8 than those present in vivo. As a result, comparison of the relative ratios may not be fully relevant, although the overall trend supports the concept that the suppression of ANGPTL3/8 LPL-inhibitory activity by ApoA5 observed in our in vitro functional assays may occur under conditions reasonably consistent with physiological regulation of TG metabolism.

Importantly, our data also reveal an additional potential mechanism by which insulin may direct the uptake of FA into adipose tissue. We previously demonstrated that insulin acts through ANGPTL8 to direct the postprandial storage of FA from food into the fat for future energy needs (11). By increasing ANGPTL8, insulin stimulates the formation of a circulating ANGPTL3/8 complex that inhibits LPL in skeletal muscle and a localized ANGPTL4/8 complex in the fat that both reduces ANGPTL4-mediated inhibition of LPL and serves to block ANGPTL3/8 inhibition of LPL in the adipose tissue. In so doing, insulin thus directs the postprandial increase of LPL-inhibitory activity to occur mainly in the skeletal muscle while ensuring that adipose tissue LPL is active so that FA are taken up mostly into the fat after feeding. By decreasing hepatocyte secretion of ApoA5, insulin might further accentuate this effect because ApoA5 blocks the LPL-inhibitory activity of the ANGPTL3/8 complex. Thus, under postprandial conditions when insulin levels are high, both the absolute amount of ANGPTL3/8 and the relative extent of the LPL-inhibitory activity of ANGPTL3/8 could be increased because insulin increases ANGPTL3/8 secretion from the liver while decreasing the hepatic secretion of its endogenous inhibitor, ApoA5, thus possibly causing less suppression of the LPL-inhibitory activity of the secreted ANGPTL3/8 complex. The reduction in ApoA5 secretion from primary human hepatocytes after insulin treatment is thus intriguing, however, further investigation will be required to show that ApoA5 levels increase during fasting and decrease when circulating insulin levels are elevated, as would occur during a hyperinsulinemic clamp.

Possible caveats with regard to our findings include that our in vitro functional experiments were performed under conditions in which it is impossible to replicate completely the environment of capillary endothelial surfaces where LPL acts in vivo to hydrolyze TGs into FAs. This is potentially important because several different proteins (including ApoC2, ApoC3, and GPIHBP1) are thought to affect LPL activity and stability and may possibly modulate the effect of ANGPTL3/8 on LPL activity (13, 14, 39, 40). In adipose tissue, LPL is transported from the underlying adipocytes across the capillary endothelial cells by GPIHPB1 and remains bound to GPIHBP1 in the capillary lumens, where GPIHBP1 appears to be important in shielding LPL and preserving its activity (39, 40). In addition, LPL activity in vivo is modulated by apolipoproteins including ApoC2, which is believed to be a stimulator of LPL activity, and ApoC3, which is thought to be an inhibitor of LPL activity (13, 14). The binding of each of these proteins to LPL therefore may affect the stability and activity of LPL as well as its interactions with the ANGPTL3/8 complex, and these interactions cannot be replicated in the in vitro functional assays used to characterize LPL activity. Along these lines, it should be noted that while we did not detect any direct ability of ApoA5 to stimulate LPL activity, our experiments were conducted in the absence of ApoC2. In contrast, Schaap *et al*. (41) demonstrated that ApoA5 could directly stimulate the LPL activity up to 2.3-fold in the presence of ApoC2 but was incapable of stimulating the LPL activity in the absence of ApoC2, indicating that some ApoA5-mediated direct stimulation of the LPL activity may be possible but only when ApoC2 is also present.

Nevertheless, despite these caveats, our data strongly suggest that the long-sought mechanism through which ApoA5 works to lower serum TG is by selectively suppressing the LPL-inhibitory activity of the ANGPTL3/8 complex. The unusual nature of this mechanism helps explain why despite being discovered 20 years ago and being recognized almost immediately as a key player in TG metabolism, the exact manner in which ApoA5 acts to decrease TG has remained stubbornly elusive. In retrospect, some hints did emerge relatively early on suggesting that the mechanism of ApoA5-mediated TG lowering was atypical. One of the first clues came with the discovery that circulating concentrations of ApoA5 were in the ng/ml range compared with other apolipoproteins such as ApoA1, which are present in the μg/ml range (30). Early suggestions that ApoA5 might act by inhibiting VLDL-TG production or directly stimulating LPL-mediated VLDL-TG hydrolysis proved difficult to reconcile with the idea that only a very small minority of VLDL particles would actually contain a molecule of ApoA5 (30).

In addition to providing the probable mechanism through which ApoA5 lowers TGs, our data also shed light on another long-standing area of investigation in the area of TG metabolism—the increases in serum TGs that occur after administration of LXR agonists. For many years, LXR agonists have been studied in preclinical models of atherosclerosis. In these models, improvements in atherosclerotic lesion burden were observed; however, administration of LXR agonists also resulted in undesirable increases in circulating TGs (42, 43, 44, 45). The increases in TGs were initially thought to be the result of increased hepatic FA synthesis and VLDL secretion and potentially surmountable. As a result, the LXR agonist BMS-852927 was advanced into clinical testing (32). In a multiple ascending dose study, however, TG elevations occurred in a dose-dependent manner with increases of up to 198% observed at day 14, suggesting that LXR agonism could cause hypertriglyceridemia in humans (32).

After considering these reports, and in light of our previous finding that insulin can stimulate secretion of ANGPTL3/8 from hepatocytes (11), we hypothesized that LXR agonists might cause hypertriglyceridemia by stimulating hepatic secretion of ANGPTL3/8. Supporting this hypothesis, LXR activation has been shown to increase ANGPTL3 and ANGPTL8 mRNA levels via SREBP-1c, while insulin activation of SREBP-1c in hepatocytes can be blocked by LXR antagonists (46, 47, 48). In addition, Inaba *et al*. (49) used ANGPTL3-deficient mice to show that ANGPTL3 can mediate hypertriglyceridemia induced by LXR activation. This observation would be consistent with LXR agonists increasing TG by increasing hepatic secretion of ANGPTL3/8 complex because no ANGPTL3/8 complex could be formed in the absence of ANGPTL3. With regard to ApoA5, LXR activation has been shown to downregulate ApoA5 mRNA levels through SREBP-1c, and insulin has been demonstrated to decrease the expression of ApoA5 via the phosphatidylinositol 3-kinase pathway (39, 50). When viewed together, these observations suggested that LXR agonists might possibly stimulate hepatocyte ANGPTL3/8 secretion while potentially decreasing ApoA5 secretion.

Using primary human hepatocytes, we demonstrated that the prototypical LXR agonist T0901317 caused a modest, yet significant, increase in hepatocyte ApoA5 secretion. After observing this, we wondered why T0901317 would increase TG, as it increases hepatic ApoA5 secretion, and ApoA5 blocks the ability of ANGPTL3/8 to inhibit LPL. We also observed, however, that T0901317 caused an almost 6-fold increase in ANGPTL3/8 secretion and that this became an almost 8-fold increase in the presence of insulin. At the same time, insulin actually decreased T0901317-stimulated secretion of ApoA5. Thus, in all likelihood, the potentially beneficial increase in ApoA5 secretion caused by T0901317 is negated by two factors. First, T0910317 stimulation in combination with physiological concentrations of insulin enhances ANGPTL3/8 secretion. Second, insulin works to abolish any T0901317-stimulated increase in the secretion of ApoA5. Therefore, it seems likely that the hypertriglyceridemia observed with LXR agonists could be due at least in part to LXR agonist-induced hepatic secretion of ANGPTL3/8.

In summary, our data shed important light on TG metabolism by showing that two key players known to be extremely important in the control of TG levels—the ANGPTL3/8 complex and the apolipoprotein ApoA5—are actually interconnected. Their unique intersection occurs through the ability of ApoA5 to selectively suppress the LPL-inhibitory activity of the ANGPTL3/8 complex. In uncovering this mode of action of ApoA5, we were able to determine that an additional mechanism through which insulin stimulates FA uptake into adipose tissue may be by decreasing hepatocyte secretion of ApoA5. Because ApoA5 is an inhibitor of ANGPTL3/8, the increased ANGPTL3/8 secreted by hepatocytes in response to insulin would be expected to have even greater LPL-inhibitory activity if ApoA5 secretion is also reduced. Similarly, we were also able to ascertain that a likely mechanism by which LXR agonists cause hypertriglyceridemia is through stimulation of hepatic ANGPTL3/8 secretion. Together, these findings provide further novel insight into the regulation of TG metabolism while at the same time suggesting that additional investigation will be required to understand more fully the molecular basis for the suppression of ANGPTL3/8-mediated LPL inhibitory activity by ApoA5.

## Data availability

All study data are contained within the article and the accompanying supplemental data file. All primary MS MRM data have been deposited at PeptideAtlas (http://www.peptideatlas.org) as follows: data identifier, PASS01645; dataset type, SRM; dataset tag, ANGPTL38_ApoA5; dataset title, SRM quantification of ANGPTL3/8, and ApoA5 proteins. All primary global proteomics data have been deposited at MassIVE (https://massive.ucsd.edu) with the following information: data identifier, MSV000086655; https://doi.org/10.25345/C5PB6F; keywords, ANGPTL38, and ApoA5.

## Supplemental data

This article contains supplemental data.

## Conflicts of interest

The authors declare that they have no conflicts of interest with the contents of this article.
