# Supplementary material for: ApoA5 lowers triglyceride levels via suppression of ANGPTL3/8-mediated LPL inhibition
Source: J Lipid Res. 2021 Mar 21;62:100068. doi: 10.1016/j.jlr.2021.100068 (PMC8079461; doi:10.1016/j.jlr.2021.100068)
Supplement: Supplemental Tables S1 and S2, Supplemental Figures S1–S20 [file mmc1.docx]

**SUPPLEMENTAL MATERIALS**

**Apolipoprotein A5 lowers triglyceride levels *via* suppression**

**of ANGPTL3/8-mediated LPL inhibition**

Yan Q. Chen^1^*, Thomas G. Pottanat^1,2^*, Eugene Y. Zhen^1^, Robert W. Siegel^1^, Mariam Ehsani^1^,

Yue-Wei Qian^1^, and Robert J. Konrad^1^**

These materials contain a complete listing of all mass spectrometry search parameters, Supplemental Tables S1-S2, Supplemental Figures S1-S20, and the accompanying Supplemental Figure Legends.

**Mass spectrometry search parameters:**

1. **Name of peak list-generating software and release version (number or date)**

The peak list was generated using an in-house software combing the search results from X! Tandem, OMSSA, and Protein pilot.

1. **Name of the search engine and release version (number or date)**

X! Tandem version 2017

OMSSA version 2.1.7

Protein Pilot 5.0.2

1. **Name of sequence database searched and release version/date**

Uniprot protein database 2015

1. **The number of entries in the database actually searched**

20345 proteins

1. **Specificity of all proteases used to generate peptides**

Trypsin

1. **The number of missed and/or non-specific cleavages permitted**

The number of missed cleavages: 2.  Non-specific cleavages are permitted.

1. **List of all fixed modifications (including residue specificity) considered**

+44.0262@C

1. **List of all variable modifications (including residue specificity) considered**

+42.0106@[,+15.9949@M,+0.9840@N,+0.9840@Q

1. **Mass tolerance for precursor ions**

10 ppm

1. **Mass tolerance for fragment ions**

X! Tandem: 0.7 Da

OMSSA: 0.8 Da

Protein Pilot: 0.6 Da.

1. **Threshold score/expectation value for accepting individual spectra**

q-value (the false discovery rate) < 0.05.

1. **Estimation of false discovery rate (FDR) and how calculated (for large datasets)**Data was searched against a reverse human database. See publication Higgs (2008, Clinical Proteomics: Methods and Protocols, vol 428, p209-230) for further details.

**Supplemental Table S1. ApoA5 peptide ions identified from mass spectrometry analysis and database search.**


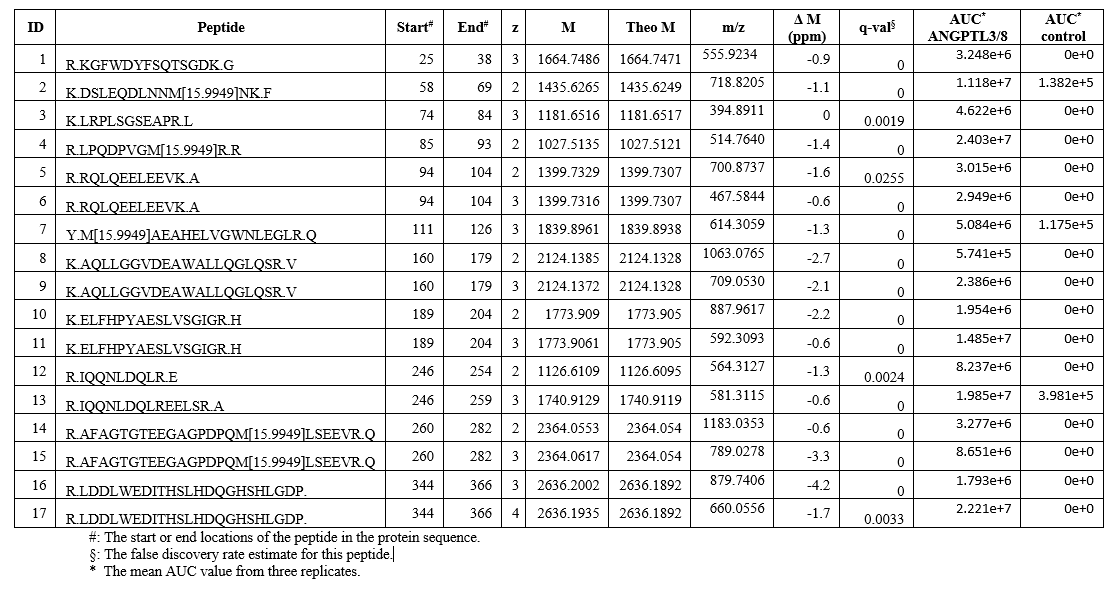


**Supplemental Table S2. ANGPTL3/8 and ApoA5 MRM peptide characteristics**


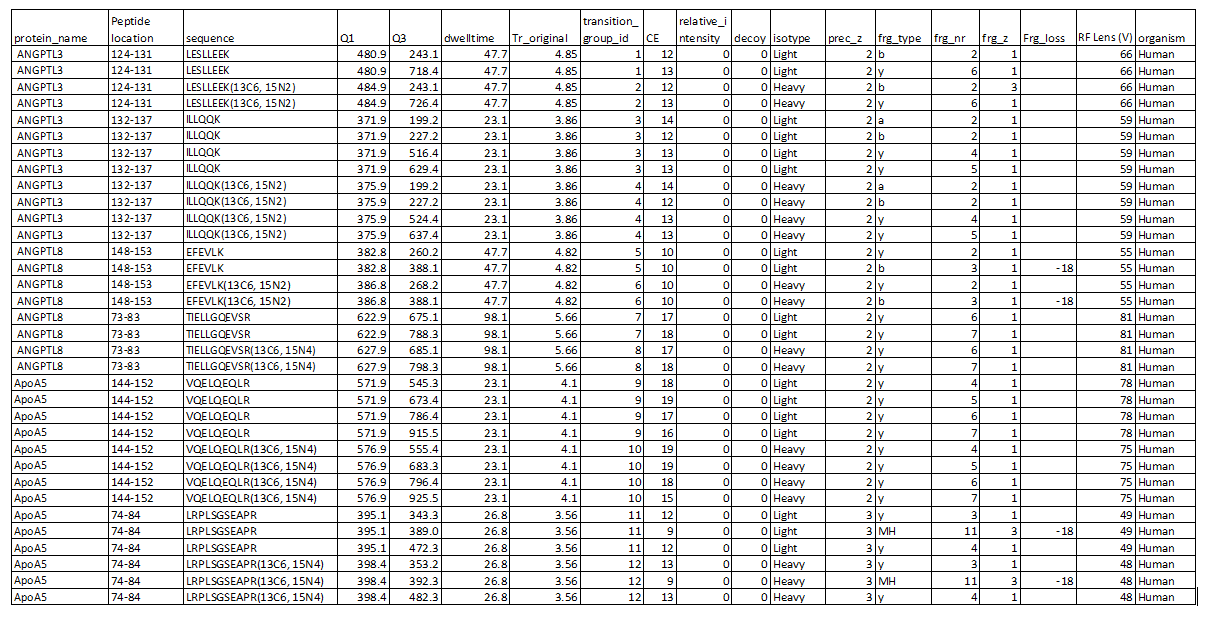


**SUPPLEMENTAL FIGURE LEGENDS**

**Supplemental Figures S1-S17: Chromatograms for ApoA5 peptide ions from ANGPTL3/8 and control samples.** The peptide ion sequence and parent m/z value are listed on top of the corresponding figures. Oxidized methionine residues were denoted with [15.9949] at the sequences. The peaks were extracted using Freestyle software with a minimum S/N threshold of 2.0 and a mass tolerance window of 2 ppm. For each ion, the ion intensity was globally normalized to the highest one in the group**.** For control samples, the peak areas were extracted within a window of ± 0.4 min of the mean retention times of the corresponding peaks detected in ANGPTL3/8 samples, as illustrated by the dash lines. The ± 0.4 min window was selected based on evaluating the maximum retention time shift of landmark ions in the samples.

**Supplemental Figures S18-S20: Chromatograms of MRM peptide ions detected in three anti-ANGPTL3/8 immunoprecipitation samples** - MRM data were quantified using Thermo Xcalibur (version 4.2.47) with ICIS peak detection algorithm. The following parameters were used for peak integration: Smoothing points: 1; S/N: 0.5; two peptides per protein were monitored. SIL peptides (0.25 pmole) were spiked into each replicate after digestion. The ratio between endogenous peptide and the corresponding SIL peptide was calculated, and the amount of protein in the samples was calculated. All analyses were performed in triplicate. The SIL peptides used for quantitation of ANGPTL3 were: 124-131 and 132-137, those used for ANGPTL8 were 73-83 and 148-153, and those used for ApoA5 were 74-84 (A5-2-2) and 144-152 (A5-1). The Y-axis shows relative abundance, and the X-axis shows retention time in minutes, with blue shading indicating the integrated area. The retention time and ion identities are listed on top of the extracted peaks.

**
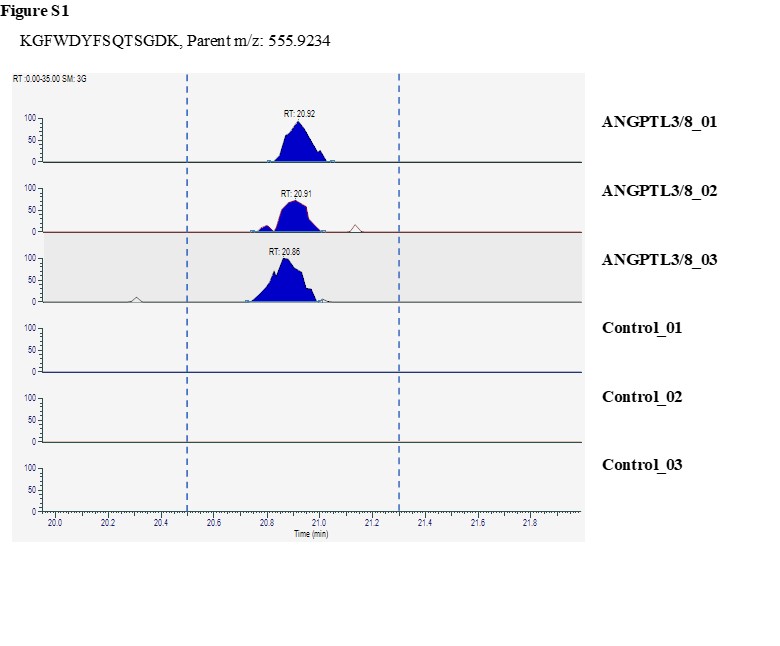
**

**
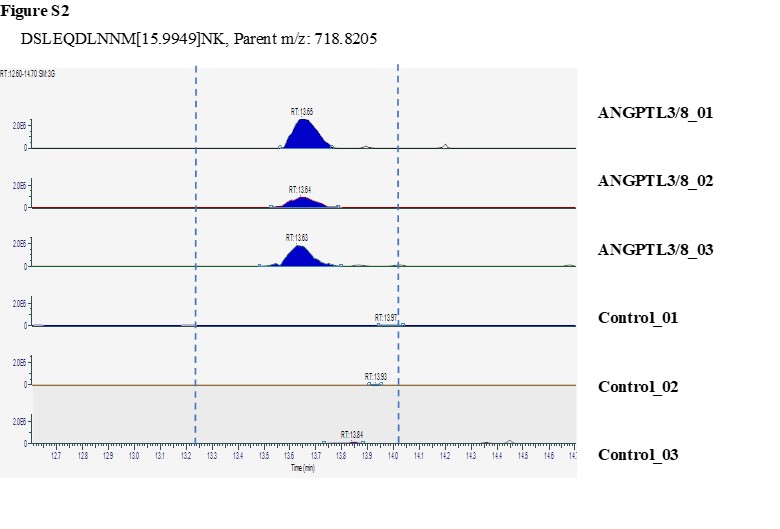
**

**
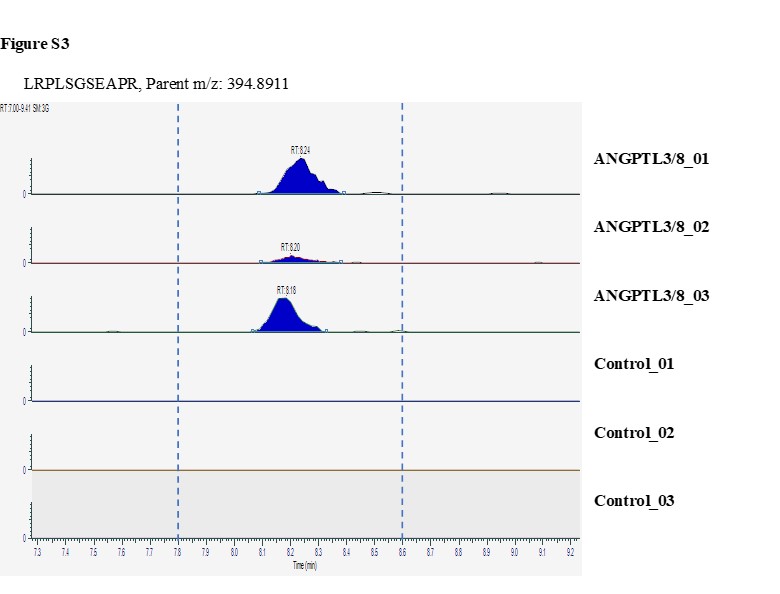
**

**
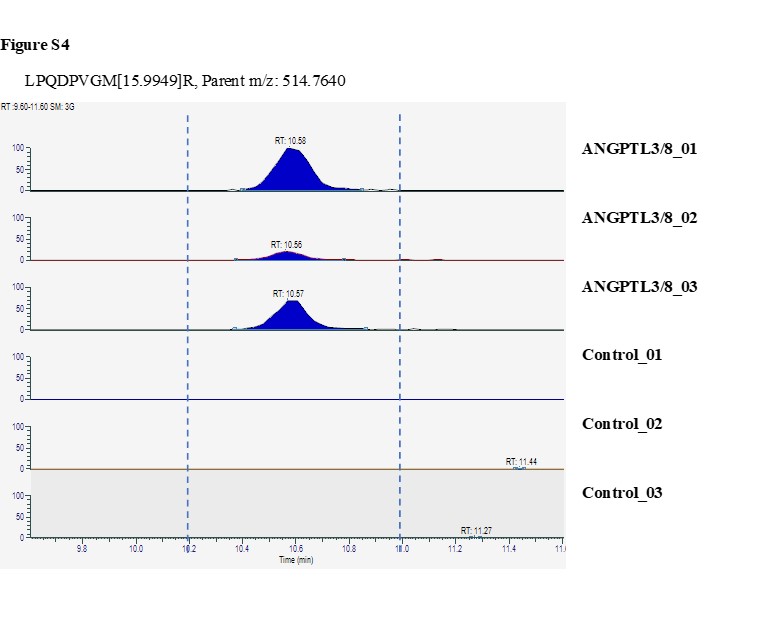
**

**
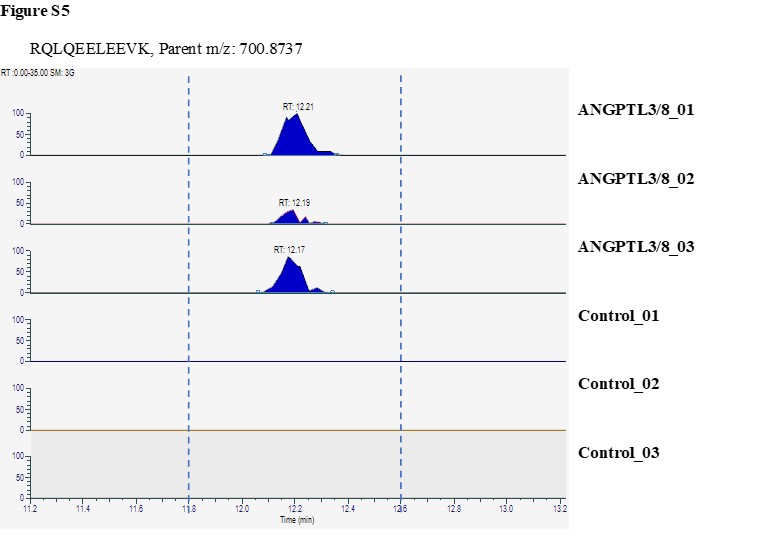
**

**
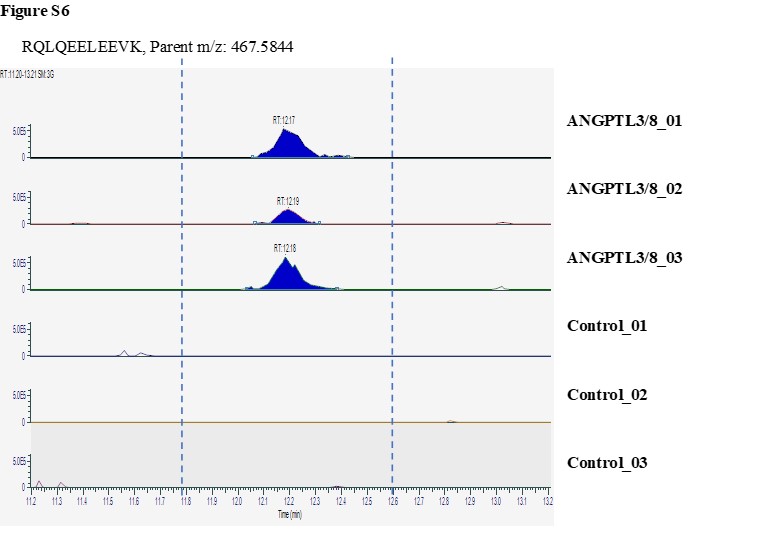
**

**
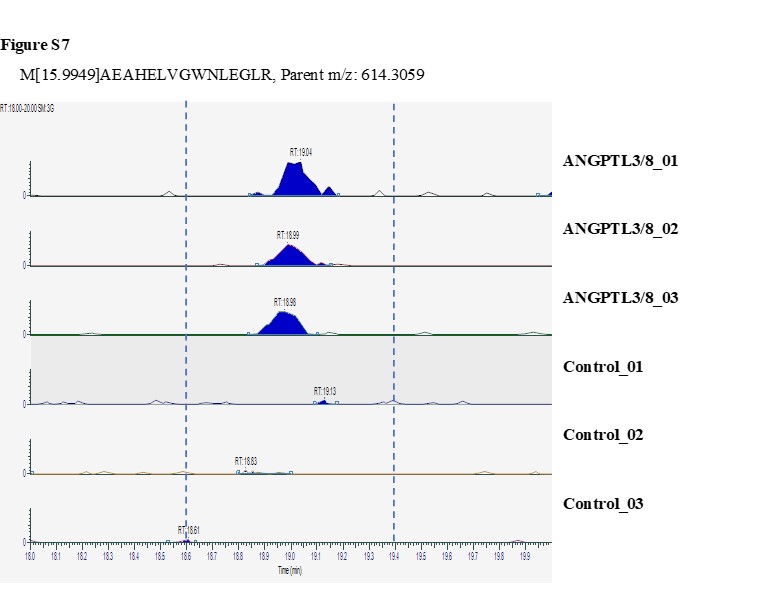
**

**
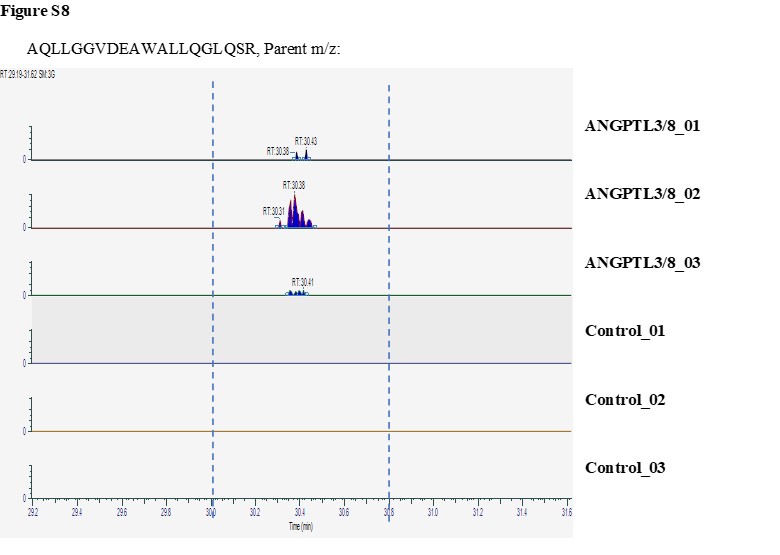
**

**
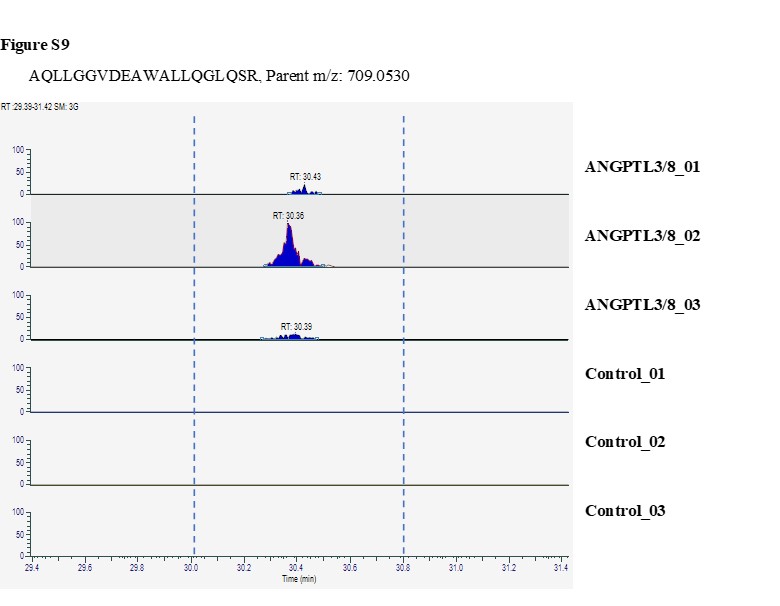
**

**
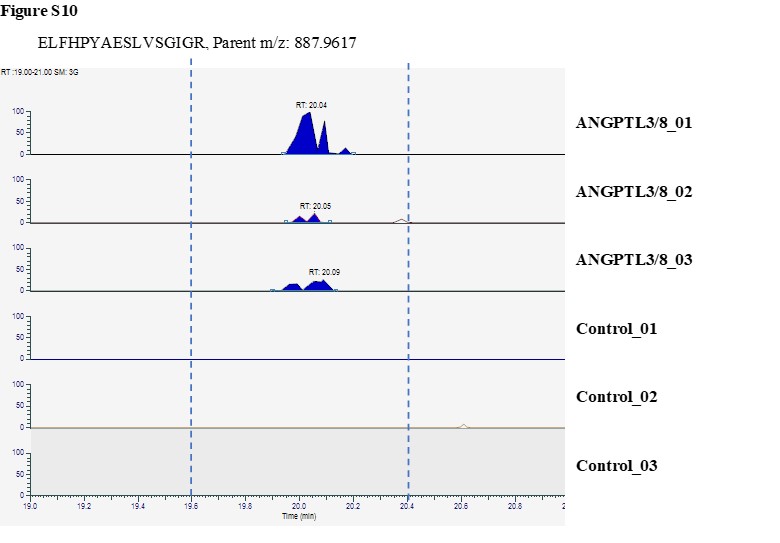
**

**
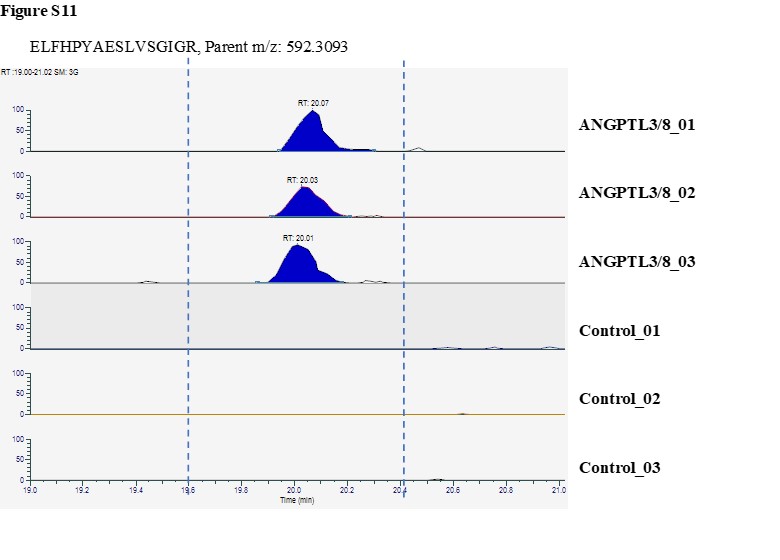
**

**
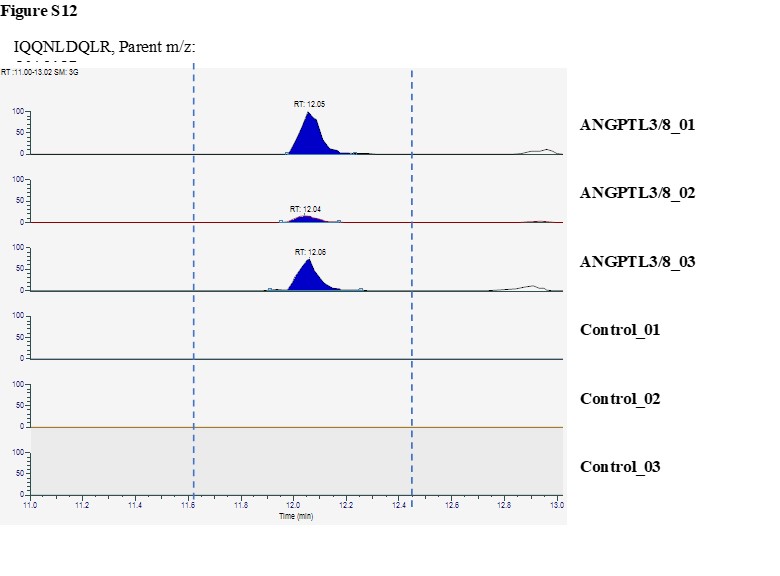
**

**
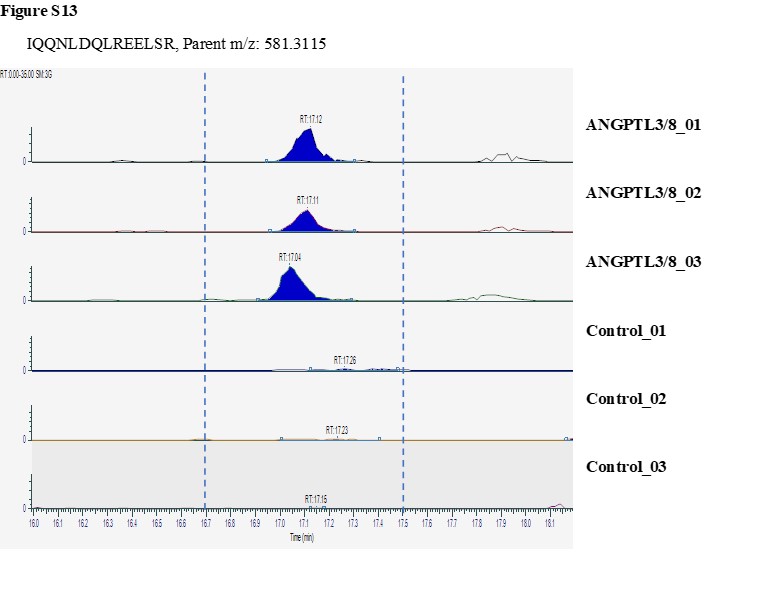
**

**
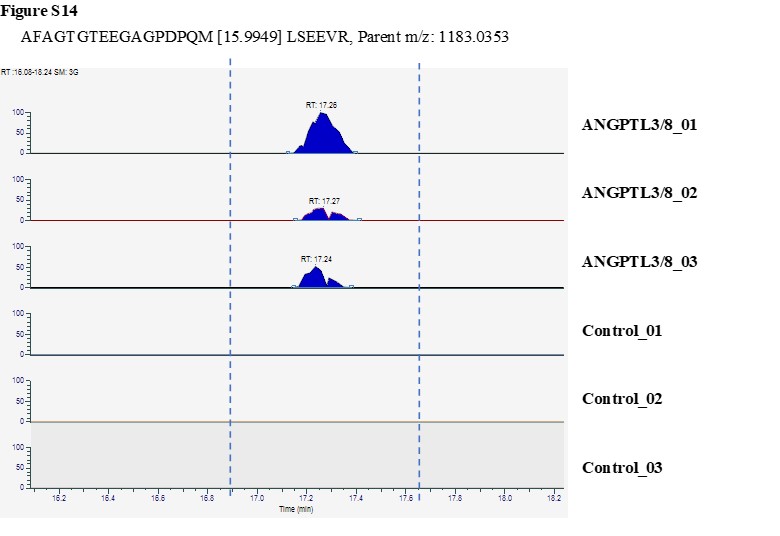
**

**
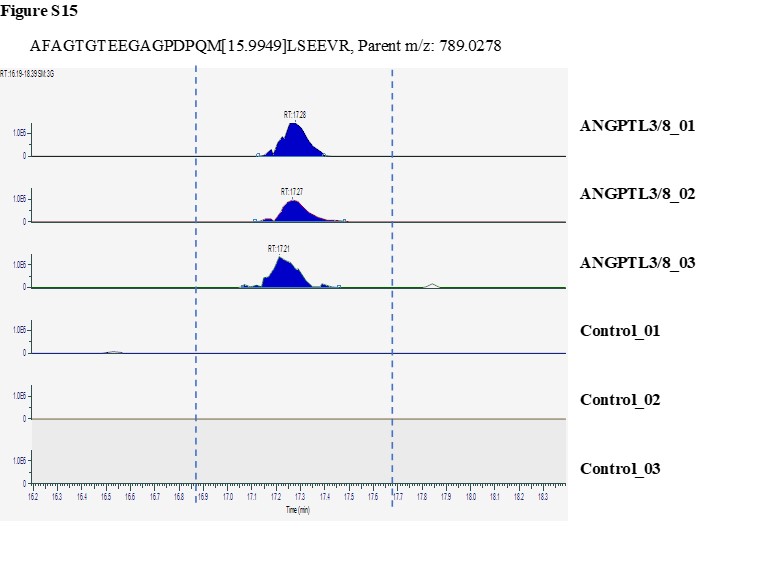
**

**
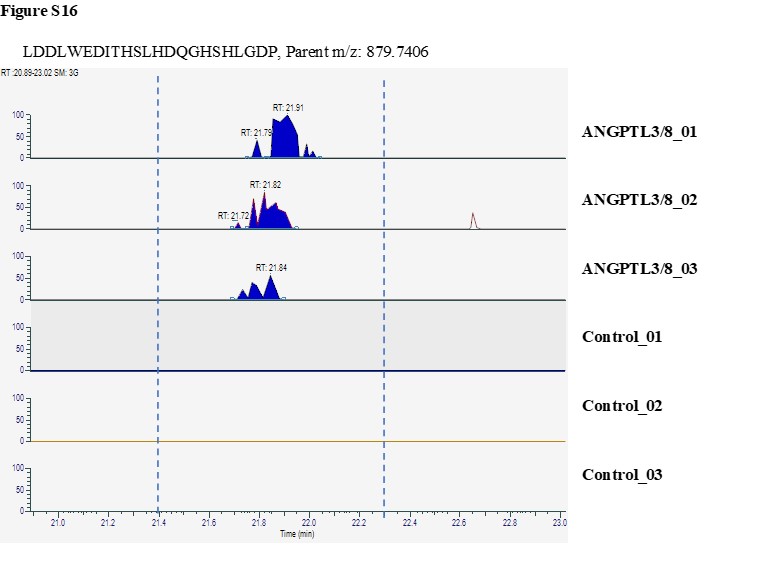
**


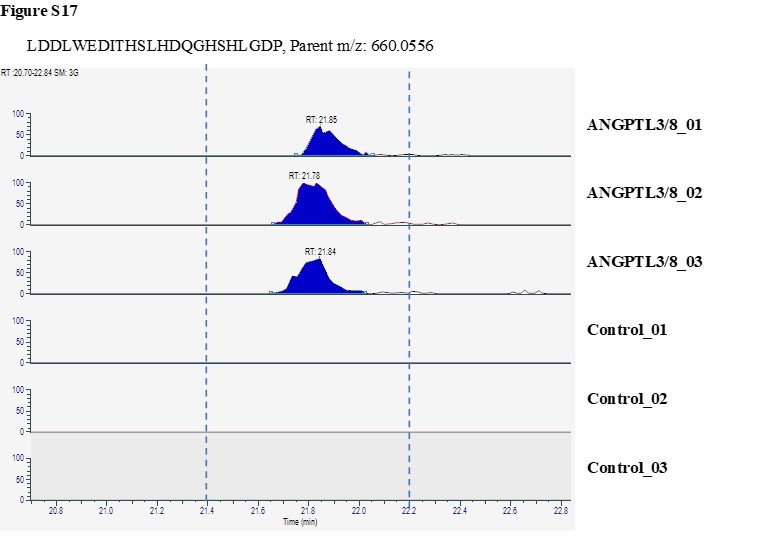


**
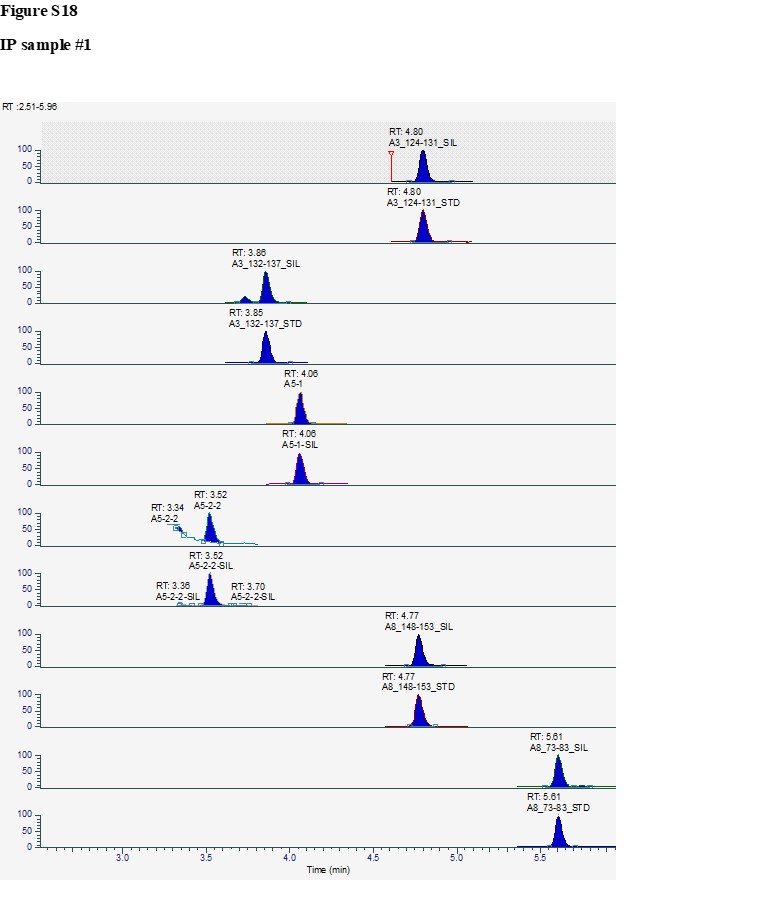
**

**
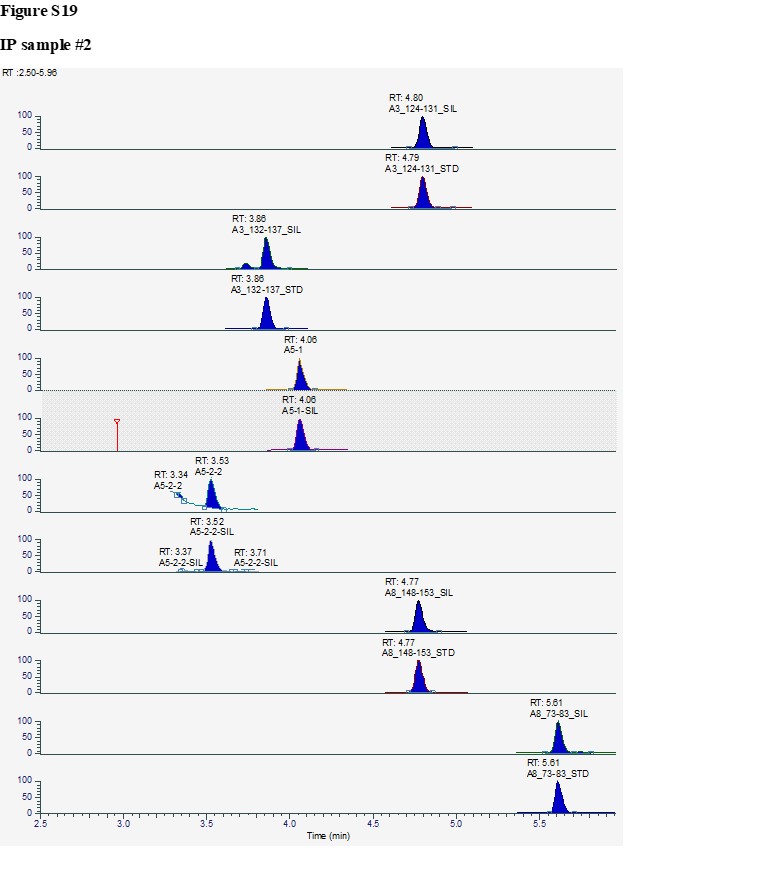
**

**
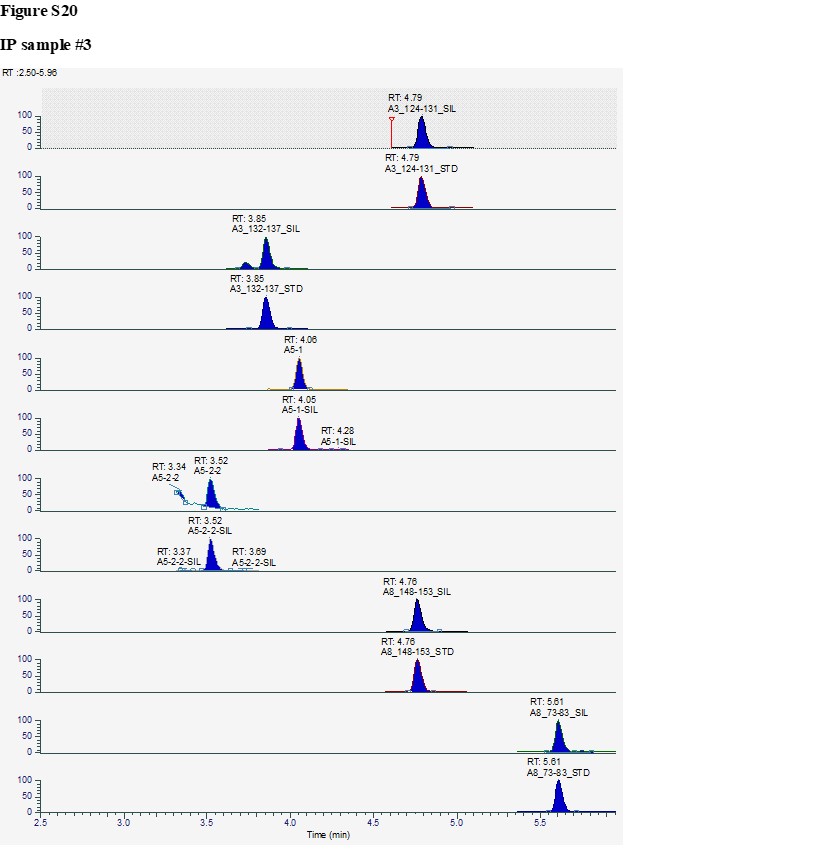
**
